# Targeting of *csgD* by the small regulatory RNA RprA links stationary phase, biofilm formation and cell envelope stress in *Escherichia coli*

**DOI:** 10.1111/j.1365-2958.2012.08002.x

**Published:** 2012-02-22

**Authors:** Franziska Mika, Susan Busse, Alexandra Possling, Janine Berkholz, Natalia Tschowri, Nicole Sommerfeldt, Mihaela Pruteanu, Regine Hengge

**Affiliations:** Institut für Biologie – Mikrobiologie, Freie Universität Berlin14195 Berlin, Germany

## Abstract

RprA is a small regulatory RNA known to weakly affect the translation of σ^S^ (RpoS) in *Escherichia coli*. Here we demonstrate that *csgD*, which encodes a stationary phase-induced biofilm regulator, as well as *ydaM*, which encodes a diguanylate cyclase involved in activating *csgD* transcription, are novel negatively controlled RprA targets. As shown by extensive mutational analysis, direct binding of RprA to the 5′-untranslated and translational initiation regions of *csgD* mRNA inhibits translation and reduces *csgD* mRNA levels. In the case of *ydaM* mRNA, RprA base-pairs directly downstream of the translational start codon. In a feedforward loop, RprA can thus downregulate > 30 YdaM/CsgD-activated genes including those for adhesive curli fimbriae. However, during early stationary phase, when *csgD* transcription is strongly activated, the synthesis of *csgD* mRNA exceeds that of RprA, which allows the accumulation of CsgD protein. This situation is reversed when *csgD* transcription is shut off – for instance, later in stationary phase or during biofilm formation – or by conditions that further activate RprA expression via the Rcs two-component system. Thus, antagonistic regulation of *csgD* and RprA at the mRNA level integrates cell envelope stress signals with global gene expression during stationary phase and biofilm formation.

## Introduction

Post-transcriptional regulation by small RNAs has been found ubiquitously in bacteria and eukaryotes. Most bacterial small RNAs base-pair to target mRNAs and thereby affect translation and/or mRNA turnover. So far, more than 80 small RNAs have been experimentally demonstrated in *Escherichia coli* (Waters and Storz, 2009). For the present study the 105 nucleotide RNA RprA is of particular interest (Fig. S1). When overproduced, RprA can activate the synthesis of σ^S^ (RpoS) (Majdalani *et al*., 2001), the stationary phase and general stress sigma subunit of RNA polymerase (RNAP) (Hengge, 2011). RprA activates translation by an anti-antisense mechanism that relieves intramolecular base-pairing of *rpoS* mRNA in the translational initiation region (TIR) (Majdalani *et al*., 2002; McCullen *et al*., 2010). Although RprA is a major small RNA (Argaman *et al*., 2001; Wassarman *et al*., 2001; Majdalani *et al*., 2002), its physiological role has remained enigmatic. In contrast to RprA overproduction, a *rprA* knockout mutation hardly affected σ^S^ levels, and additional targets of RprA have not been identified.

Two lines of evidence suggested that RprA may have a function in the context of stationary phase and biofilm control: (i) the only known RprA target, *rpoS* mRNA, encodes the master regulator of stationary phase gene expression, which also activates the expression of biofilm components such as adhesive curli fimbriae (summarized in Hengge, 2011), and (ii) RprA expression is activated by the RcsC/RcsD/RcsB two-component signal transduction pathway, which is involved in biofilm maturation (Majdalani and Gottesman, 2005).

RcsC, RcsD and RcsB constitute a phosphorelay signalling pathway, which in enteric bacteria affects several stress responses, biofilm formation and capsule synthesis. The Rcs system is activated by numerous stimuli that include cell envelope perturbations, high osmolarity, desiccation, low temperature and growth on surfaces, but the underlying mechanisms are poorly understood. The membrane-bound histidine sensor kinase RcsC can activate the response regulator RcsB by phosphotransfer via the HPT protein RcsD. In the absence of appropriate stimuli, RcsC acts as a phosphatase for RcsB and thereby actively downregulates the output of the pathway (for a comprehensive review, see Majdalani and Gottesman, 2005). As a DNA-binding transcription factor, phosphorylated RcsB directly activates not only *rprA* (Fig. S1), but a number of genes involved in stress responses and biofilm formation, including genes involved in the production of the biofilm matrix polysaccharide colanic acid (Gottesman *et al*., 1985; Ferrières and Clarke, 2003; Hagiwara *et al*., 2003; Francez-Charlot *et al*., 2005; Castanie-Cornet *et al*., 2010; Krin *et al*., 2010). On the other hand, RcsB can directly repress *flhDC* which encodes the master regulator for flagella expression (Francez-Charlot *et al*., 2003). Overall, the Rcs system can thus downregulate motility, maintain or increase cellular levels of σ^S^ and modulate biofilm formation (Majdalani and Gottesman, 2005).

Global gene expression patterns are similar in stationary phase and in biofilms (Schembri *et al*., 2003; Beloin *et al*., 2004), and σ^S^ is required for the expression of many genes involved in biofilm formation (Hengge, 2011). One of these genes is *csgD*, which encodes an important early biofilm regulator (Römling, 2005). Apart from σ^S^-containing RNAP, *csgD* transcription also depends on the diguanylate cyclase YdaM and the transcription factor MlrA, which are expressed from σ^S^-dependent genes as well (Römling *et al*., 1998; Brown *et al*., 2001; Weber *et al*., 2006; Pesavento *et al*., 2008). Among the CsgD-regulated target genes are the structural genes for adhesive curli fimbriae (*csgBA*) and *yaiC* which encodes a diguanylate cyclase involved in cellulose biosynthesis (Römling, 2005). For simplicity, this complex transcriptional cascade is referred to as the ‘curli control cascade’ in the following (summarized in Fig. S2).

CsgD is also regulated by the Rcs phosphorelay system, but in contrast to the Rcs-mediated input into σ^S^ control, CsgD is negatively controlled (Vianney *et al*., 2005). Downregulation of CsgD and curli expression can be triggered by the expression of YmgB, a small protein that acts via the Rcs pathway (Tschowri *et al*., 2009). The observation that this effect of YmgB could be suppressed by knocking out the *rprA* gene (Fig. S3) suggested that CsgD regulation by RcsB occurs indirectly via the RcsB-controlled small RNA RprA.

We therefore decided to study the molecular function of RprA in *csgD* and curli regulation in closer detail. Here we report that RprA directly interacts with the 5′-regions of both *csgD* mRNA and *ydaM* mRNA. Thereby, RprA can directly reduce *csgD* mRNA levels and translation and, indirectly via YdaM, reduce transcription of *csgD*. However, during entry into stationary phase, strong expression of excess *csgD* mRNA overcomes inhibition by RprA, which allows the synthesis of CsgD protein and the activation of more than 30 genes including those for curli fimbriae. Yet, activation of the Rcs two-component system resulting in only about fourfold induction of RprA is sufficient to completely shut off the expression of CsgD and its target genes. Overall, *csgD* mRNA and the small RprA RNA cooperate to inversely control a large CsgD/RprA regulon that co-ordinates early stationary phase and biofilm gene expression with the Rcs cell envelope stress response.

## Results

### Mutations in the Rcs phosphorelay system and RprA oppositely affect the expression of *rpoS* and genes in the curli control cascade

**Fig. 1:**
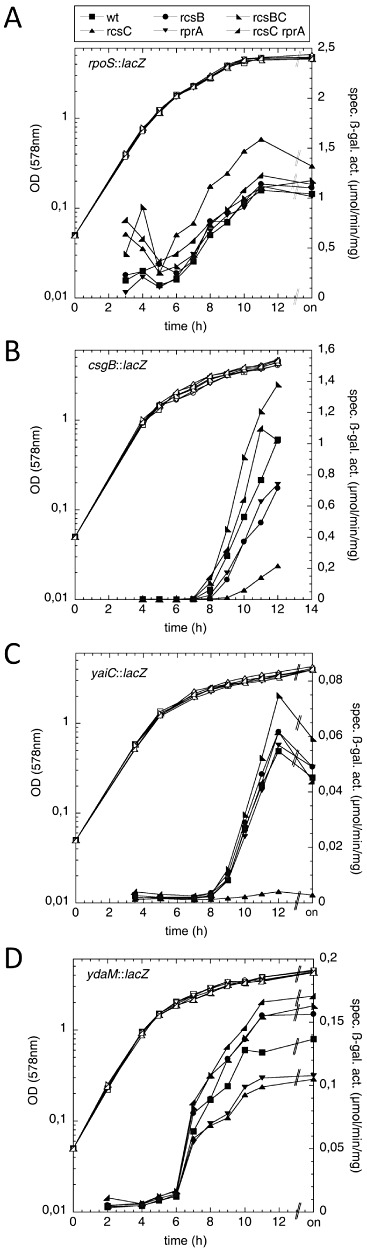
Differential control of genes in the σ^S^/YdaM/CsgD control cascade by the Rcs two-component system and the small RNA RprA. Expression of single copy translational *lacZ* reporter fusions in *rpoS* (A), the two CsgD-controlled genes *csgB* (B) and *yaiC* (C) and the intermediate regulatory gene *ydaM* (D) was assayed (for the functions of these genes in the curli control cascade, see Fig. S2). Derivatives of strain W3110 carrying fusions and *rcs/rprA* knockout mutations as indicated were grown in LB at 28°C. OD_578_ (open symbols) and specific β-galactosidase activities (closed symbols) were determined throughout the growth cycle.

In order to gain a first overview of the influence of the Rcs/RprA system in the curli control cascade, we systematically analysed the effects of single and double knockout mutations in the *rcs* genes and *rprA* on translational *lacZ* reporter fusions in various regulatory and target genes in this cascade (Fig. 1). Mutations in *rcsC* and *rcsB* actually have opposite effects, since in the absence of RcsC-dependent phosphatase activity, RcsB can be activated via ‘cross-talk’ from other unidentified phosphodonors (Majdalani and Gottesman, 2005). Consistent with activation of *rpoS* expression by RprA (Majdalani *et al*., 2001; 2002), *rpoS* expression was somewhat increased in the *rcsC* mutant, which could be suppressed by secondary mutations in either *rcsB* or *rprA* (Fig. 1A).

By contrast, two downstream target genes of the σ^S^/CsgD control cascade, *csgB* and *yaiC*, showed the opposite pattern. These genes were strongly downregulated in the *rcsC* mutant, and this inactivation was completely relieved by knocking out *rcsB* or *rprA* (Fig. 1B and C). Among the regulatory genes in the curli control cascade, *ydaM* was similarly affected (but the effects were weaker than for *csgB* and *yaiC*; Fig. 1D) while *mlrA* expression was unaltered (data not shown). These data indicated that downstream of the positively RprA-regulated master regulator σ^S^, there must be at least one negatively regulated RprA target in the curli control cascade. *ydaM* seemed one candidate, but the much stronger effects of the *rcs/rprA* mutations on the CsgD targets *csgB* and *yaiC* pointed to *csgD* expression as a possible major RprA target. Moreover, this was also in line with suppression of the effect of the YmgB/Rcs pathway on CsgD and curli expression by a mutation in *rprA* (Tschowri *et al*., 2009 and Fig. S3).

### RprA downregulates the expression of the biofilm regulator CsgD

**Fig. 2:**
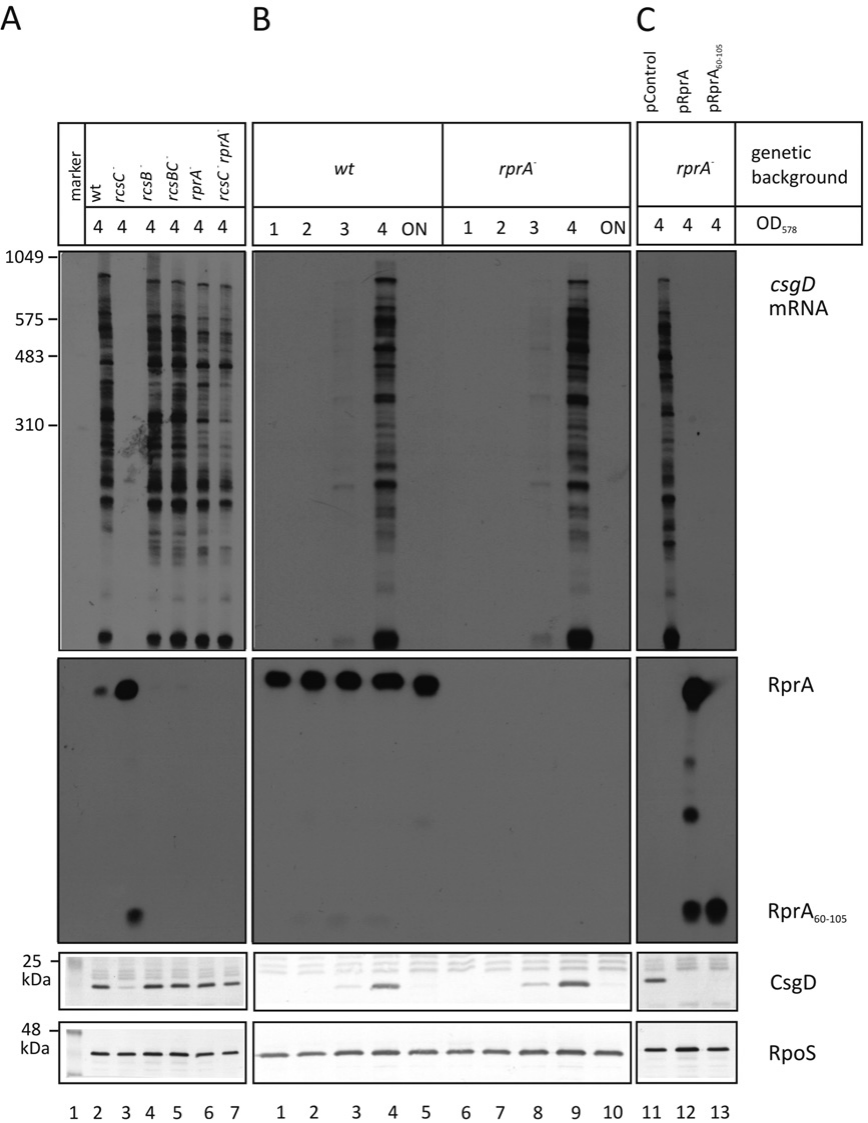
Cellular levels of *csgD* mRNA, RprA, CsgD and σ^S^ in strains carrying mutations in the Rcs/RprA system or overproducing RprA. Derivatives of strain W3110 carrying the mutations and/or RprA-overproducing plasmids as indicated were grown in LB at 28°C. The OD_578_ during sampling is mentioned in the figure. *csgD* mRNA and RprA levels were determined by Northern blot analysis (using a 5′-end *csgD* probe; 5S rRNA was always used as an internal control; data not shown). CsgD and σ^S^ (RpoS) levels were assayed by immunoblotting. Note that *csgD* mRNA is by far the major transcript derived from the *csgDEFG* operon with larger transcripts also encompassing the following genes being hardly visible under the conditions used here. Film exposure for the detection of plasmid-encoded RprA was approximately 10-fold shorter than for chromosomally encoded RprA.

Therefore we analysed how the same *rcs*/*rprA* mutations affected the cellular levels of *csgD* mRNA, RprA and the CsgD and σ^S^ (RpoS) proteins during entry into stationary phase (Fig. 2A). In the *rcsC* mutant, *csgD* mRNA as well as CsgD protein were strongly reduced, whereas RprA accumulated to higher levels than in the parental strain (under these conditions, also a previously reported degradation product of RprA became apparent, i.e. RprA_60–105_; Argaman *et al*., 2001). The long ‘ladder’ of incomplete *csgD* mRNA fragments are 5′-end fragments since the Northern blot probe used here was complementary to nucleotides −148 to +90 of *csgD* mRNA. These data show that increased expression of RprA in the *rcsC* mutant correlated with strongly reduced expression of *csgD*. σ^S^ levels, however, were hardly affected (Fig. 2A), consistent with the relatively weak effects seen with the *rpoS::lacZ* translational fusion (Fig. 1A).

**Fig. 3:**
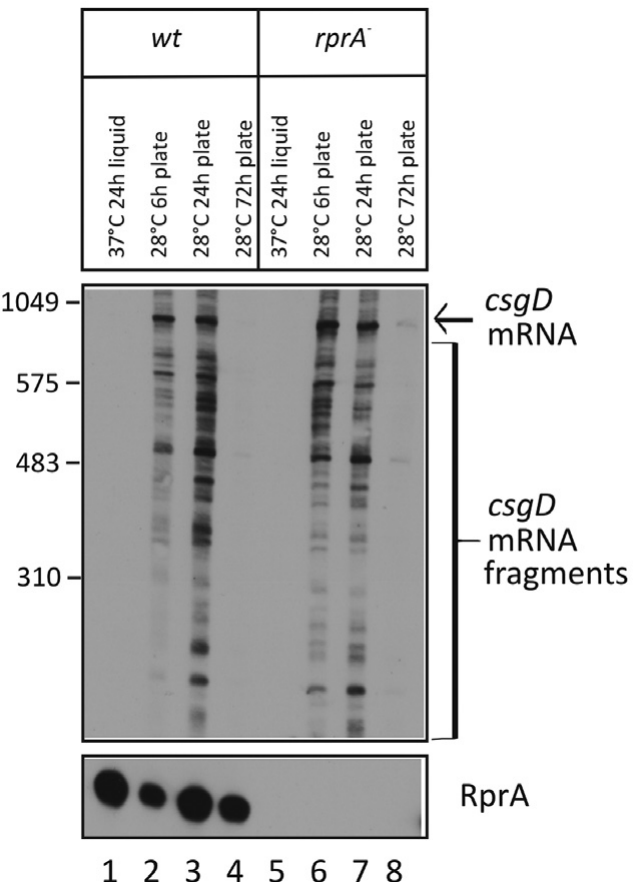
Cellular levels of *csgD* mRNA and RprA in long-term colonies on agar plates. Strain W3110 and its *rprA*::*kan* mutant derivative were grown in liquid LB at 37°C and as patches on LB agar plates at 28°C. Sampling in liquid was at an OD_578_ of 4.0, from the plates at the times after inoculation as indicated. *csgD* mRNA and RprA levels were determined by Northern blot analysis.

When assayed along the growth cycle (Fig. 2B), growth phase-dependent expression of CsgD was clearly apparent. *csgD* mRNA and traces of CsgD protein appeared around an OD_578_ of 3, and were maximal around an OD_578_ of 4, i.e. during early stationary phase. Notably, *csgD* mRNA as well as CsgD protein were no longer present later in stationary phase, i.e. in an overnight culture, whereas RprA as well as σ^S^ persisted (Fig. 2B). Disappearance of *csgD* mRNA and CsgD protein equally occurred in a *rprA* mutant background, and is therefore not caused by RprA but probably by a shutdown of *csgD* transcription and possibly CsgD proteolysis (Fig. 2B). With cells growing for several days as patches on agar plates, i.e. in surface biofilms, a similar pattern was observed. Both *csgD* mRNA and RprA were present after 6 and 24 h of growth, but *csgD* mRNA was no longer found after 72 h, whereas RprA continued to be present (Fig. 3).

When RprA was constitutively overproduced from a plasmid, *csgD* mRNA as well as CsgD protein were absent (Fig. 2C). When overproduced, also the truncated form of RprA (RprA_60–105_), which corresponds to the highly conserved region of RprA (Fig. S1), was sufficient to downregulate *csgD* mRNA and CsgD protein (Fig. 2C).

Overall, these data demonstrate that RprA can downregulate *csgD* expression at the mRNA level, even when only moderately overproduced (such as in the *rcsC* mutant).

### RprA reduces expression of CsgD and YdaM also when expressed from ectopic promoters

**Fig. 4:**
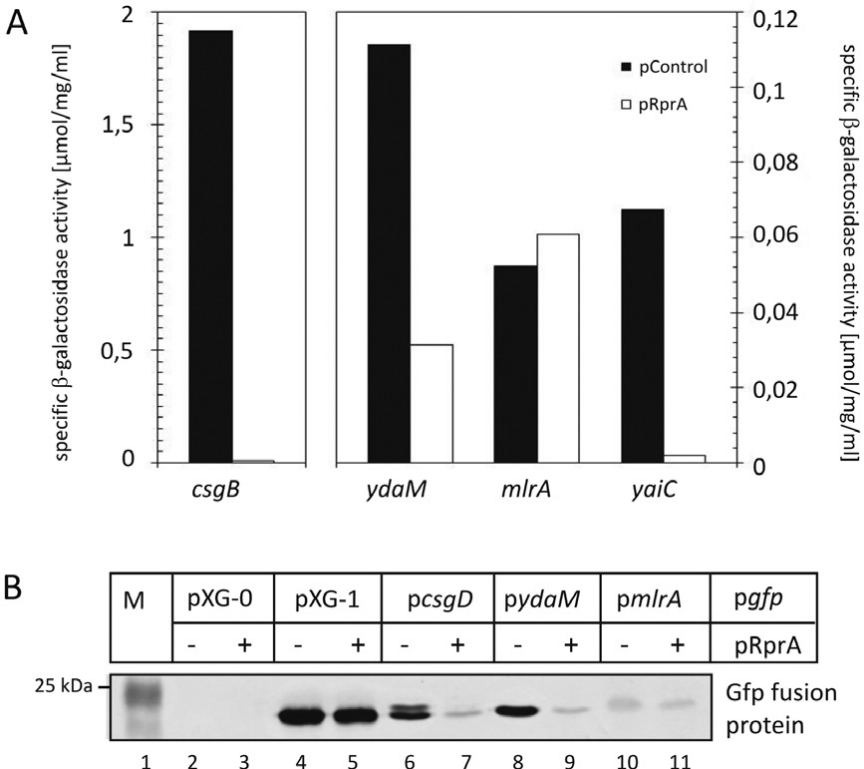
RprA overproduction reduces the expression of *lacZ* and *gfp* reporter fusions to several genes in the curli control cascade. W3110 carrying single copy *lacZ* fusions to the genes indicated (under the control of their natural promoters; A) or MC4100 carrying low copy number plasmid-encoded *gfp* fusions (under the control of the ectopic P_LtetO_ promoter; B) in combination with either pRprA or the corresponding vector were grown in LB at 28°C (A) or 37°C (B) to an OD_578_ of 4.0. Specific β-galactosidase activities (A) and Gfp fusion protein levels (immunoblots shown in B) were determined. pXG-0 and pXG-1 are control plasmids without *gfp* and containing *gfp* only respectively.

In order to study a putative direct interaction of RprA with its target(s) in the curli control pathway, we used a convenient test system in which both the small RNA of choice and its putative target gene are constitutively expressed from ectopic promoters on compatible plasmids. The target gene is fused to *gfp*, which allows to use the level and/or activity of the Gfp hybrid protein as a simple readout of the system (Urban and Vogel, 2007). Using an ectopic promoter for expression of these *gfp* fusion constructs (with the same 5′-ends as present in the wild-type mRNAs) uncouples target gene expression from putative complex indirect effects of RprA, such as RprA acting on *rpoS* and *ydaM* (Fig. 1A and D), with σ^S^ activating *ydaM* transcription, and both activating *csgD* transcription (Fig. S2). Direct effects on target mRNAs, however, are maintained. As shown for *csgD* and *ydaM* (Fig. 4B), the *gfp* fusions reproduced the effects of RprA overproduction seen with translational single copy *lacZ* fusions in *ydaM* and the CsgD target genes *csgB* and *yaiC* (Fig. 4A), whereas the other regulatory gene in the curli control pathway, *mlrA*, was not affected. These data suggested that RprA may directly act on the mRNAs of *csgD* and *ydaM*.

### RprA interferes with translation and reduces *csgD* mRNA levels by direct interaction

**Fig. 5:**
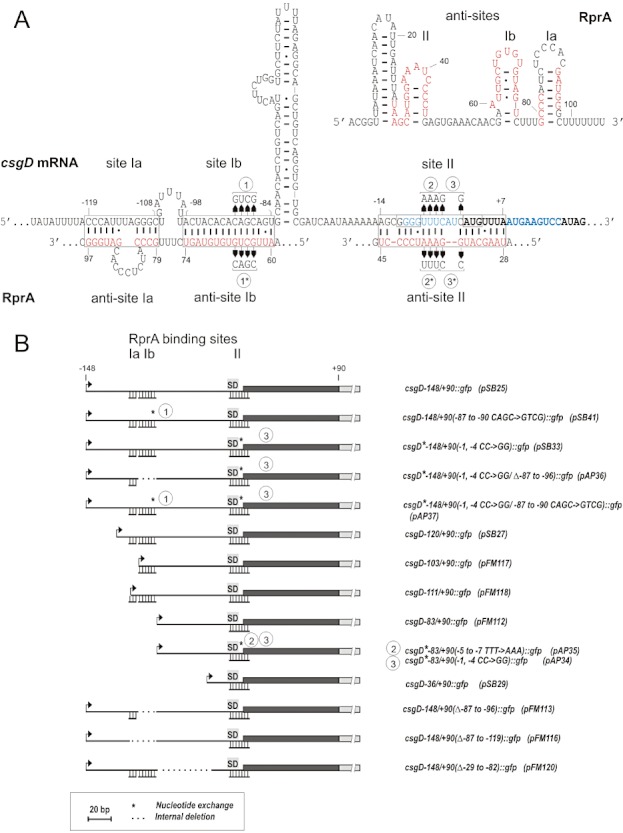
The 5′-region of *csgD* mRNA and RprA, their putative interaction sites and relevant genetic constructs. A. The part of *csgD* mRNA showing complementarity to RprA (sites Ia, Ib and II) and including the putative stem-loop between nucleotides −83 and −28 [interaction was predicted with the *RNAhybrid2.2* program (Rehmsmeier *et al*., 2004); numbering refers to the translational start site on *csgD* mRNA] as well as full-size RprA in its putative folded structure are shown [secondary structures predicted by Mfold (Zuker, 2003); numbering is from the 5′ to the 3′ end]. Regions in RprA likely to interact with *csgD* mRNA are highlighted in red (anti-sites Ia, Ib and II). Blue nucleotides indicate a region in *csgD* mRNA that can form a small stem-loop structure that includes the TIR. Numbers in circles refer to mutated nucleotides as indicated. B. Positions of deletions and point mutations that were isolated on pSB25, which carries the −148 to +90 region of *csgD* inserted precisely behind the P_LtetO_ promoter and fused to *gfp*. Numbers in the names of the fusion constructs refer to the nucleotide sequence in (A).

The wild-type *csgD* transcript as well as the *csgD::gfp* mRNA used here both contain the 148 nucleotide 5′-untranslated leader region (5′-UTR). Predictions of interaction between RprA and the *csgD* leader revealed extensive complementarity in two regions termed sites I and II (Fig. 5A). Region I, which extends from positions −119 to −84 of *csgD* mRNA and is interrupted into two half-sites (Ia and Ib) by a small loop region (−107 to −99), is complementary to a region between nucleotides 60 and 97 in RprA (termed anti-sites Ia and Ib). *csgD* mRNA region II (from −14 to +7) overlaps with the TIR and is complementary to nucleotides 28 to 45 of RprA (anti-site II). Regions I and II on *csgD* mRNA are separated by a stretch of 70 nucleotides that was shown to form a long stem-loop structure (Holmqvist *et al*., 2010) (Fig. 5A).

**Fig. 6:**
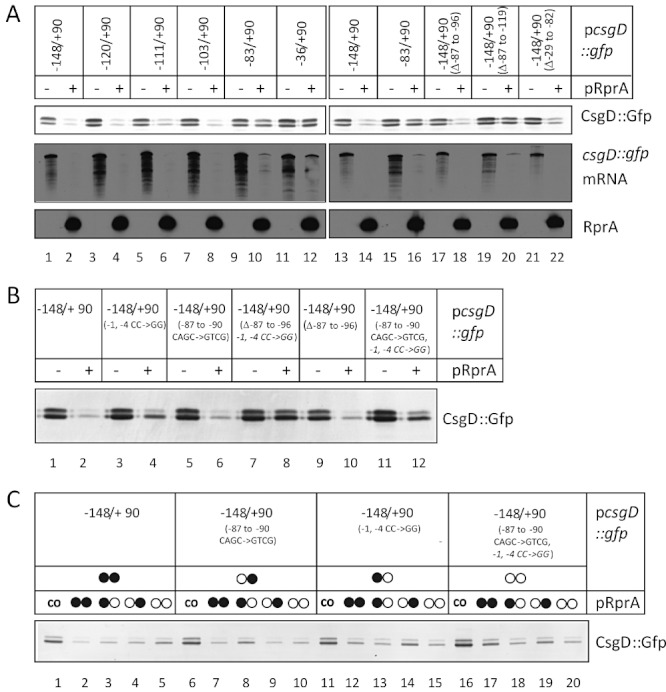
Genetic identification of regions in the 5′-UTR/TIR of *csgD::gfp* mRNA relevant for RprA-mediated regulation and evidence for direct interaction of the two RNAs. In an *rprA*::*kan* derivative of MC4100, effects of RprA overproduction were assayed in the presence of 5′- and internal deletions (A) and point mutations (B) in the 5′-UTR/TIR of *csgD::gfp* mRNA. In (C) all possible combinations of point mutations in either site I or site II (or both) of *csgD*::*gfp* mRNA with RprA carrying either one or both complementary exchanges in anti-site I or II are shown. Allelic changes and their locations are symbolized by pairs of black (wild-type sequence) or white circles (mutant sequences), with the left and right circles indicating the status of site I/anti-site I and site II/anti-site II respectively. ‘co’ stands for the control plasmid (pJV300, which expresses a small nonsense RNA). Cells were grown in LB at 37°C to an OD_578_ of 4.0. mRNA and protein levels of the *csgD*::*gfp* reporter fusion were determined by Northern and immunoblot analyses respectively.

In order to sort out how these regions of complementarity might contribute to RprA-mediated regulation of *csgD*, we isolated a series of 5′- and internal deletions in the 5′-UTR as well as point mutations in regions I and II on the *csgD::gfp* constructs (Fig. 5B). With these constructs, we assayed both mRNA and protein levels in the absence and presence of the RprA-overproducing plasmid (Fig. 6). The 5′- as well as precise internal deletions (Fig. 6A, left and right panel respectively) demonstrated that deleting site Ia alone (construct −103/+90, left panel lanes 7 and 8) or Ib alone (construct −148/+90 Δ−87 to −96, right panel, lanes 17 and 18) did not relieve strong downregulation of *csgD*::*gfp* mRNA and CsgD::Gfp protein by RprA. However, eliminating the entire site Ia/Ib was sufficient to generate increased amounts of CsgD::Gfp protein (constructs −83/+90 and −36/+90, left panel lanes 9, 10 and 11, 12 respectively, and −148/+90 Δ−87 to −119, right panel lanes 19, 20). *csgD*::*gfp* mRNA levels were also increased, although not to the level seen in the absence of the RprA plasmid, suggesting that translation is reduced only when transcript levels fall below a certain threshold. Deleting the long stem-loop region between −29 and −82 of *csgD* mRNA, which leaves intact sites Ia/Ib and II, did not affect the ability of RprA to downregulate *csgD*::*gfp* mRNA and CsgD::Gfp protein (last two lanes in Fig. 6A).

As site II contains the translation initiation site, its role in a putative interaction with RprA could not be studied by simple deletion. We therefore isolated precise exchanges in nucleotides that are predicted to interact with RprA but that are not part of either the Shine-Dalgarno (SD) site or the initiation codon [CC(−1,4)GG, see Fig. 5A]. On its own, this exchange in site II did not affect RprA-mediated downregulation of CsgD::Gfp (Fig. 6B). However, when combined to a 4 bp exchange in site Ib [CAGC(−87to−90)GTCG] or a deletion of the entire site Ib (Δ−87 to −96), which by themselves also had no effects, translational inhibition was relieved (Fig. 6B). This indicated that regions I and II both contribute to, but to some extent are also functionally redundant in RprA-mediated regulation.

In order to genetically demonstrate a direct interaction between RprA and *csgD*::*gfp* mRNA, we isolated changes in RprA that are complementary to the point mutations in site Ib and site II, i.e. to CAGC(−87to−90)GTCG and CC(−1,4)GG (mutations 1 and 3 in Fig. 5A). Combining these mutant constructs (as well as their wild-type counterparts) with all mutant/wild-type versions of RprA revealed a clear suppression pattern (Fig. 6C). Consistent with the data described above, any single mutation alone (either in the *csgD* construct or in RprA), i.e. a single mismatch in putative base-pairing, did not significantly relieve RprA-mediated downregulation of *csgD*::*gfp* expression. Combining any two mutations (either both in *csgD* or in RprA or ‘crosswise’ combinations of mutations in both) that would result in two putative mismatches relieved downregulation of *csgD*::*gfp*. However, combinations of two mutations in *csgD* and RprA that would restore the putative base-pairing also restored downregulation of *csgD*::*gfp*. Moreover, the effects of both *csgD* mutations together could be successively suppressed by combining them with either one or both complementary mutations in RprA (Fig. 6C). These data are a clear indication of direct base-pairing between *csgD*::*gfp* mRNA and RprA both in sites I and II.

This result was further corroborated by suppression obtained with another mutation in site II (TTT(−5to−7)AAA; mutation 2 in Fig. 5A) and the complementary exchange in RprA (Fig. S4). In addition, we observed that this TTT/AAA exchange resulted in elevated CsgD::Gfp protein levels (in the absence of the RprA plasmid; Fig. S4). This mutation in site II actually disrupts a small stem-loop structure which overlaps with the TIR (blue nucleotides in Fig. 5A, structure shown in Fig. S4) and reduces translation as recently shown (Holmqvist *et al*., 2010).

In conclusion, these data indicate that: (i) an extensive region in the 5′-UTR of *csgD* mRNA (sites Ia/Ib and II) is involved in a partially redundant manner in RprA-mediated downregulation of *csgD* expression; (ii) RprA interacts directly with *csgD* mRNA at these sites; (iii) this interaction does not only reduce translation but also *csgD* mRNA levels; and (iv) the long stem-loop region between −29 and −83 of *csgD* mRNA is not involved in the action of RprA and does not seem to generally affect *csgD* expression under the conditions tested here.

### RNase III, RNase E and Hfq are not essential for RprA-mediated downregulation of *csgD* mRNA

Small RNAs that reduce target mRNA levels usually do so by stimulating endonucleolytic attack, either by allowing double strand specific RNase III to cleave in the duplex region which allows further rapid decay of the resulting fragments (Arraiano *et al*., 2010), by specifically recruiting the target mRNA to a Hfq-RNase E complex (Morita *et al*., 2005; Pfeiffer *et al*., 2009; Caron *et al*., 2010) or by allowing RNase E to access RNA that is not protected by ribosomes when translation initiation is inhibited by the sRNA (Massé*et al*., 2003; Chen *et al*., 2004; Afonyushkin *et al*., 2005; Wagner, 2009). In order to test whether RNase III, RNase E or Hfq are involved, we assayed the effect of RprA on *csgD* mRNA in the corresponding *rnc*, *rne* and *hfq* mutants. While a specific small *csgD* mRNA fragment was degraded in the RprA-overexpressing strains in an RNase III-dependent manner, RprA-mediated downregulation of full size and all other fragments of *csgD* mRNA was independent of RNase III (Fig. S5). Since RNase E is essential, we used a temperature-sensitive *rne* mutant (Goldblum and Apririon, 1981). At the non-permissive temperature, the RprA effect on *csgD* mRNA was slightly less pronounced but still clearly visible (Fig. S6), suggesting that RNase E may somewhat contribute to, but is not essential for RprA-mediated downregulation of *csgD* mRNA. Also a mutation in *hfq* relieved downregulation of *csgD* by RprA only slightly (Fig. S7), suggesting that Hfq plays an auxiliary role in the formation of the RprA/*csgD* mRNA complex, but is not essential for downregulating *csgD* mRNA.

### RprA reduces YdaM expression by directly interacting with *ydaM* mRNA

**Fig. 7:**
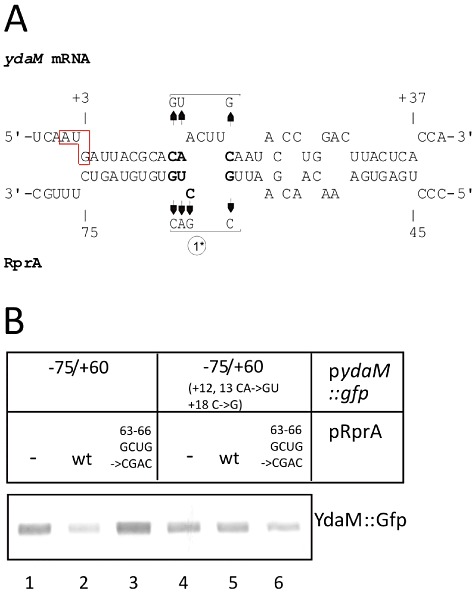
Phenotypic suppression by complementary mutations in *ydaM::gfp* mRNA and RprA provides evidence for direct interaction of the two RNAs. A. Partial complementarity between the region immediately downstream of the start codon of *ydaM* mRNA and the central region of RprA as well as the compensatory nucleotide exchanges introduced are shown [interaction was predicted with the *RNAhybrid2.2* program (Rehmsmeier *et al*., 2004); numbering refers to the translational start site on *ydaM* mRNA]. B. YdaM::Gfp protein levels were determined in *rprA*::*kan* derivatives of MC4100 carrying combinations of plasmids expressing wild-type or mutated *ydaM*::*gfp* and *rprA* as indicated. Cells were grown and analysed as described in the legend to Fig. 6.

The data shown in Fig. 4 suggested that *ydaM* mRNA might be another direct target for RprA. A region immediately downstream of the *ydaM* start codon (nucleotides +3 to +37) shows several stretches of complementarity to the RprA sequence (nucleotide 45 to 75; this region includes anti-*csgD* site Ib; see Figs 5A and 7A). A 4 bp exchange in this region of RprA (designated as mutation ‘1*’) not only reduces base-pairing to *csgD* mRNA, but is also predicted to affect this putative base-pairing to *ydaM* mRNA. This mutation in RprA indeed relieved downregulation of *ydaM::gfp* expression by RprA (compare lanes 1 to 3 in Fig. 7B). This effect was suppressed by introducing the complementary exchanges in *ydaM::gfp* (Fig. 7; compare lanes 4 to 6 in Fig. 7B). This direct suppression indicates a direct interaction between *ydaM* mRNA and RprA. Moreover, this implies that RprA can affect CsgD expression not only directly, but also indirectly via YdaM.

### CsgD and RprA tightly cooperate in controlling global gene expression

Our identification of the *csgD* and *ydaM* mRNAs as novel direct targets for RprA also raised the question whether RprA has even more targets. In addition, since CsgD is itself a transcriptional regulator, CsgD-controlled genes may constitute a significant or even major subset of the RprA regulon.

**Fig. 8:**
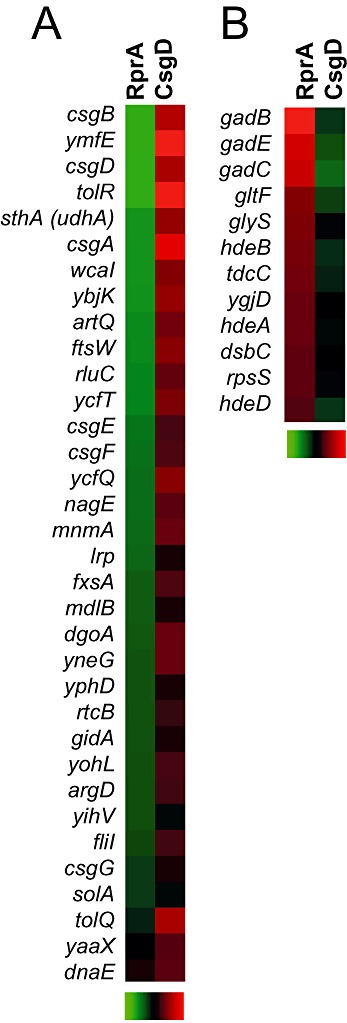
The RprA and CsgD regulons during entry into stationary phase. *E. coli* K-12 strain W3110 and its derivatives were grown in LB at 28°C to an OD_578_ of 4 and genome-wide transcriptome analysis was performed as detailed in *Experimental procedures*. The following strains were compared: for the RprA regulon, *rcsC*::*cat* versus *rcsC*::*cat rprA*::*kan*; for the CsgD regulon: *csgD*^+^ versus Δ*csgD*. Ratios of differential gene expression (listed in Table S1) were transformed into a ‘heat map’ as described in *Experimental procedures*, with (A) showing genes under negative control (green) by RprA and positive control (red) by CsgD, and (B) showing oppositely regulated genes; genes were ordered by the magnitude of RprA dependence.

Using microarray-based transcriptome analysis, we identified the regulons controlled by RprA and CsgD and the extent to which these overlap. During entry into stationary phase (in LB at 28°C), when *csgD* mRNA physiologically accumulates to high levels, comparing *csgD^+^* and Δ*csgD* strains revealed a large regulon of predominantly positively CsgD-regulated genes including the curli operon *csgBA* (Fig. 8, Table S1). While RprA is also expressed under these conditions (Fig. 2), knocking out *rprA* did not reveal any genes with significant differential regulation on the microarrays (data not shown), consistent with the very minor effects also on the expression of *rpoS* and known CsgD target genes (see Figs 1 and 2). These results indicate that when wild-type cells just enter into stationary phase and are not subject to any other stress, the ongoing RprA synthesis is not sufficient to significantly reduce mRNA levels of *csgD* or affect any other putative target genes.

However, what would be the consequences for global gene expression if RprA was further induced in response to some conditions that may further activate the Rcs system? In order to simulate this situation we again used the *rcsC* mutant, in which RcsB is activated and RprA shows approximately fourfold higher expression (Fig. 2A), and compared this strain to the *rcsC rprA* double mutant. This allowed us not only to identify RprA-regulated genes (with a majority being under negative RprA control), but revealed an almost complete and inverse overlap between the RprA and CsgD regulons, i.e. negatively RprA-regulated genes were found to be under positive control of CsgD and vice versa (Fig. 8, Table S1). This pattern was even apparent for genes that were just weakly regulated by one regulator and below the threshold of significance (set to ratios < 0.5 or > 2) for the other (Table S1). The only clear exception to this pattern seemed to be *gadE* and the *gadBC* operon, which exhibited positive regulation by RprA and only very weak negative effects of CsgD. This suggested that RprA may be able to positively target *gadE*, which encodes a regulator that activates *gadBC* expression. *rpoS* and *ydaM* did not show up as RprA target genes in the microarray data, probably because higher levels of RprA in the *rcsC* mutant do not affect these mRNAs strongly enough (Fig. 1).

Taken together, these data allow several conclusions: (i) RprA-regulated genes expressed during entry into stationary phase are largely identical to CsgD targets and thus constitute a common ‘CsgD/RprA regulon’ inversely controlled by these two regulators; (ii) when wild-type cells enter into stationary phase, RprA is expressed but functionally silent and the large set of genes under positive control of *csgD* is expressed; (iii) this set of genes as well as *csgD* itself are negatively regulated by RprA, when RprA is expressed at only moderately higher levels (as in the *rcsC* mutant); (iv) while most effects on global gene expression of RprA are thus linked to altered levels of *csgD* mRNA and/or CsgD protein, *gadE* seems to be RprA-regulated in a *csgD*-independent manner and therefore may be an additional direct target for RprA besides *csgD*, *ydaM* and *rpoS*.

## Discussion

### A regulator of regulators – the small regulatory RNA RprA modulates the expression of several globally acting transcription factors

With this study we have identified *csgD* and *ydaM* as novel direct targets for the small regulatory RNA RprA (Figs 3–6). In addition, our data provide preliminary evidence that also *gadE* (Table S1) is controlled by RprA in a *csgD*-independent and possibly direct manner. RprA regulation of *ydaM* sets up a feedforward loop (FFL), which combines direct downregulation of *csgD* by RprA at the mRNA level with indirect downregulation via YdaM, which encodes a diguanylate cyclase producing c-di-GMP that is essential for *csgD* transcription initiation (Weber *et al*., 2006). This FFL arrangement may enhance and speed up *csgD* downregulation under sudden stress conditions incompatible with proper assembly of the amyloid curli fibres.

The other RprA targets, i.e. *rpoS*, *csgD* and possibly *gadE*, all encode globally acting transcription factors. With σ^S^ (the product of the *rpoS* gene) activating the transcription of *csgD* as well as of *gadE* in complex feedforward cascades, these regulators are the key components of a regulatory network that co-ordinates global gene expression during stationary phase, biofilm formation and stress conditions (summarized in Hengge, 2011). Within this transcriptional network, RprA introduces differential control at the mRNA level by maintaining or further stimulating the expression of σ^S^ and GadE while downregulating that of YdaM/CsgD when required.

### The small regulatory RNA RprA directly binds to *csgD* mRNA, interferes with translation and reduces the cellular level of *csgD* mRNA

Our finding that RprA also downregulates *csgD* when expressed from an ectopic promoter (Figs 4, 6 and S4) as well as phenotypic compensation by complementary nucleotide exchanges in the two RNAs (Figs 6C and S4) indicate that RprA directly binds *csgD* mRNA. This interaction involves separate regions of extensive RprA complementarity on *csgD* mRNA (Fig. 5A): (i) site I (−119 to −84) consists of two parts, Ia and Ib, separated by a small loop region; (ii) site II (−14 to +7) overlaps with the TIR and is clearly required for translational inhibition by RprA (Figs 5 and S4). These segments act in a partially redundant manner, as introducing deletions or point mutations in either site Ia, Ib or site II *alone* did not reduce the effects of RprA on *csgD* mRNA or CsgD protein. However, introducing the same lesions in two of the three regions (no matter in which combinations) alleviated translational inhibition, indicating that RprA binding was no longer strong enough to efficiently interfere with ribosome entry into the translational start site (Fig. 6). At the *csgD* transcript level, even deleting the entire Ia/Ib region (−83/+90, −36/+90) seemed to relieve the inhibitory effect of RprA only partially, while the effect at the Gfp fusion protein level was clearer. It should be noted, however, that with these constructs, *full-size* transcripts (which are the only ones that can give rise to the full-size proteins shown in the immunoblots) were clearly present despite RprA overproduction, in contrast to their absence in the constructs that carried the complete 5′-UTR or lesions in one of the three regions only.

The 5′-UTR of *csgD* mRNA can also form a long stem-loop structure which separates RprA-binding sites Ia/b and II (Fig. 5A). A precise deletion of this stem-loop region, which leaves intact the complete regions of RprA complementarity, did not affect RprA action on *csgD* mRNA, nor did it affect translation or mRNA levels at all (Fig. 6). Yet, this stem-loop region is bound and dissolved by two closely related small regulatory RNAs, OmrA/B (Holmqvist *et al*., 2010). Overproduction of OmrA/B interferes with translational initiation on *csgD* mRNA by an unknown mechanism that acts at a distance (the target site and the SD region are about 50 nucleotides apart); yet, Holmqvist *et al*. also observed that the stem-loop region *per se* is not required for translation. Moreover, OmrA/B overproduction did not seem to alter *csgD* mRNA levels. Thus, *csgD* mRNA can be directly bound at non-overlapping sites by two distinct small RNAs, RprA and OmrA/B, both of which reduce translation. However, the molecular mechanisms of action seem different, as only RprA interacts directly with the SD region and also strongly reduces the mRNA level.

### How does RprA RNA reduce the cellular level of *csgD* mRNA?

RprA can impose kind of a ‘death kiss’ on *csgD* mRNA, as it does not only interfere with its translation but also reduces its cellular level so efficiently that *csgD* mRNA can hardly be detected upon even moderate overproduction of RprA (Fig. 2). This downregulation is directly linked to the interaction of these two RNAs and reduced *csgD* mRNA translation, since it occurs also with *csgD*::*gfp* mRNA expressed from an ectopic promoter, and lesions in *csgD* mRNA and/or RprA that relieve translational inhibition do also relieve transcript downregulation (Figs 6 and S4). Even in the absence of RprA, *csgD* mRNA levels are actually sensitive to a variation in the rate of translation, as can be seen with the −83/+90 construct (Fig. S4). Here, the TTT(−5to−7)AAA mutation, which eliminates a small translation-inhibitory stem-loop structure (Holmqvist *et al*., 2010) that overlaps with the *csgD* TIR region (blue nucleotides in Fig. 5A, structure shown in Fig. S4C), results in an about twofold increase in CsgD::Gfp protein levels and several fold higher *csgD::gfp* mRNA level (Fig. S4A).

In principle, at least two molecular mechanisms could account for RprA-mediated regulation of *csgD* mRNA levels: RprA binding to the 5′-part of *csgD* mRNA and inhibition of *csgD* translation initiation may (i) stimulate mRNA turnover and/or (ii) induce premature termination of transcription. Since site I of the 5′-UTR of *csgD* mRNA, which features a particularly long stretch of complementarity to RprA, emerges from RNAP right after transcription initiation, RprA may bind to the *nascent csgD* transcript already and stimulate its co-transcriptional endonucleolytic cleavage and decay. However, although small RNA-dependent modulation of transcript stability is relatively common (see reviews by Arraiano *et al*., 2010; Caron *et al*., 2010), neither the double-strand specific RNase III nor RNase E is essential for RprA to downregulate *csgD* mRNA, although the RprA effect was somewhat less pronounced in the absence of active RNase E (Figs S5 and S6). This suggests that RNase E contributes to some extent to the effect of RprA on *csgD* mRNA as it does in the effects of the small RNAs RyhB or MicC on their respective target mRNAs (Massé*et al*., 2003; Chen *et al*., 2004; Afonyushkin *et al*., 2005; Wagner, 2009). However, stimulating RNase E-dependent degradation does not seem to be the only mechanism by which RprA acts on *csgD* mRNA.

In addition, rapid binding of RprA to nascent *csgD* transcripts may also affect transcript elongation. RNAP and the immediately following, i.e. ‘leading’ ribosome can directly interact via NusG protein which results in a mechanistic coupling of transcription and translation (Burmann *et al*., 2010; Proshkin *et al*., 2010; Roberts, 2010). In the absence of such a physically linked ribosome, the elongated RNAP is prone to ‘backtracking’ and premature termination of transcription by termination factor Rho, an effect long known as polarity (Adhya *et al*., 1974; Franklin, 1974; Richardson *et al*., 1975). Thus, excess RprA, by disfavouring ribosome binding to nascent *csgD* mRNA emerging from RNAP, may increase the frequency of premature termination of nascent *csgD* transcripts. By contrast, when *csgD* is expressed in excess over RprA, the majority of nascent *csgD* transcripts could pick up a ‘leading’ ribosome and these transcripts would be elongated and translated.

RprA-induced degradation as well as premature transcriptional termination of nascent *csgD* transcripts could equally produce the trail of incomplete *csgD* 5′-fragments visible on the Northern blots (Fig. 2). Moreover, the two processes are not necessarily mutually exclusive. Finally, since both processes would result from an inhibition of translational initiation, our data also suggest another mechanistic detail. Downregulation of *csgD* mRNA was also observed with overproduction of the RprA(60–105) fragment, which can base-pair to region Ia/Ib only, but not directly to the TIR (Figs 2 and S5). This is consistent with the proposal by Holmqvist *et al*. (2010) that a region upstream of the *csgD* TIR contributes to translational initiation as a transient ribosome loading site (Unoson and Wagner, 2007). This proposal was based on the finding that OmrA/B, which inhibits translation initiation of *csgD* mRNA, binds to the long stem-loop region right next to site Ia/Ib and therefore also upstream of the TIR (Holmqvist *et al*., 2010).

### RprA and CsgD mRNA set up an RNA network that underlies the co-ordination of stationary phase, biofilm formation and the cell envelope stress response

When *E. coli* cells enter into stationary phase or during initial biofilm formation (Figs 2 and 3), *csgD* transcription is strongly activated, which allows *csgD* mRNA as well as CsgD protein to accumulate. While RprA is synthesized in parallel, its knockout has hardly any effect on the expression of *rpoS*, *ydaM*, *csgD*, *csgB* and *yaiC* (Figs 1 and 2), nor does it affect genome-wide gene expression (as observed in our microarray analyses). This suggests that either RprA has no other targets under these conditions or that *csgD* mRNA is a high-affinity target whose strong expression and binding to RprA prevents the latter from affecting other target RNAs. The region of RprA complementarity to *csgD* mRNA (Fig. 4) is significantly longer than for *rpoS* mRNA (Majdalani *et al*., 2002) or *ydaM* mRNA (Fig. 7). It is also longer than required for downregulation of *csgD* mRNA and inhibition of *csgD* translation (see above). This suggests that *csgD* mRNA is a priority target, which may ‘trap’ RprA similar to trapping of the small RNA MicM (also termed ChiX, SroB or RybC) by a precisely regulated inhibitory RNA (Figueroa-Bossi *et al*., 2009; Overgaard *et al*., 2009). Notably, the many incomplete *csgD* mRNA fragments visible on the Northern blots (see e.g. Fig. 2) correspond to the 5′-region of *csgD* mRNA, indicating that most of these fragments can contribute to trapping RprA. Overall, early stationary phase is thus a ‘CsgD-ON’ state characterized by high levels of *csgD* mRNA and the synthesis of CsgD protein and a ‘silencing’ of RprA activity even though RprA is actually synthesized.

Our microarray data (Fig. 8, Table S1) show that in this ‘CsgD-ON’ state, a CsgD/RprA regulon is activated that consists of more than 30 genes which are not expressed in a Δ*csgD* mutant or when *csgD* expression is downregulated by enhanced RprA synthesis (in the *rcsC* mutant). The molecular mechanisms for activation of these genes can be rather different. In some cases, which include the *csgBA* operon (Römling *et al*., 2000; Ogasawara *et al*., 2011), CsgD protein can directly control transcription. The mRNAs of some other genes, however, could be direct targets for RprA, which ‘escape’ this control as long as RprA remains trapped by highly expressed *csgD* mRNA. Some genes of the CsgD/RprA regulon may even be targets for additional *csgD* mRNA-binding small RNA(s) (such as OmrA/B) that could also be sequestered by highly expressed *csgD* mRNA. In this scenario, the *csgD* gene actually has a *dual* role in global gene expression – its mRNA acts in a RNA network that prevents RprA and perhaps other small regulatory RNAs from affecting their other target mRNAs, while its protein product acts as a transcription factor.

In this RNA network, the status of expression of the entire CsgD/RprA regulon must be highly sensitive to changes in the *ratio* of expression rates for *csgD* and *rprA*. Either upregulation of RprA expression or downregulation of *csgD* expression (or both at the same time) should result in switching from a ‘CsgD-ON/RprA-OFF’ to a ‘CsgD-OFF/RprA-ON’ state. What is the physiological context in which this may occur? The Rcs system, which activates RprA expression, responds to a variety of cell envelope perturbations (Majdalani and Gottesman, 2005). It is easy to imagine that the massive assembly of curli fimbriae, which is typical for early stationary phase cells, becomes detrimental and has to be shut down when cells have to cope with envelope stress. Another condition, where the ratio of expression for *csgD* mRNA and RprA shifts in favour of RprA is later in stationary phase (Figs 2 and 3). Here, both *csgD* mRNA as well as CsgD protein disappear, while RprA expression continues and also σ^S^ protein remains present. That RprA is not required for *csgD* mRNA and CsgD to disappear (Fig. 2) suggests that *csgD* transcription is inactivated and CsgD protein is degraded. Upon shutdown of *csgD* mRNA synthesis, RprA would now be free to bind to other potential targets, i.e. certain mRNAs synthesized late in stationary phase. The same may apply to other small RNAs bound by *csgD* mRNA such as OmrA/B (see above). Overall, what seems to be emerging here, is a fine-tuned network of directly interacting mRNAs and small RNAs, in which every player can be a regulator as well as a target. The architecture and physiological impact of this non-hierarchical regulatory RNA network and its multiple connections with the hierarchical transcriptional network of *E. coli* will have to be elucidated in future studies.

## Experimental procedures

### Bacterial strains and growth conditions

The strains used in this study are derivatives of the *E. coli* K-12 strains W3110 (Hayashi *et al*., 2006) and MC4100 (Casadaban, 1976). The otherwise isogenic strains BL322 and BL321 carry the *rnc* wild-type allele encoding endoribonuclease RNase III and a non-functional *rnc* mutant allele respectively (Studier, 1975). The isogenic strains N3433 and N3431 carry the *rne* wild-type allele and an *rne*^ts^ allele (*rne-3071*) respectively; the latter expresses a thermolabile endoribonuclease RNase E that is rapidly inactivated upon shift to the non-permissive temperature of 43°C (Goldblum and Apririon, 1981). The *hfq*::omega knockout allele was previously described (Tsui *et al*., 1994). The construction of mutant alleles, plasmids and single copy *lacZ* and plasmid-encoded *gfp* reporter fusions is described in detail in *Supporting information* (including primers shown in Table S2).

Cells were grown in LB medium (*Miller, 1972*) under aeration at 28°C (strains with wild-type control of *csgD*) or 37°C (strains in which *csgD* is under ectopic promoter control). Antibiotics were added as detailed in *Supporting information*. Growth was monitored by measuring the optical density at 578 nm (OD_578_).

### SDS-PAGE and immunoblot analysis

Sample preparation for SDS-PAGE and immunoblot analysis were performed as described previously (Lange and Hengge-Aronis, 1994). Three, five or ten micrograms of cellular protein was applied per lane. Polyclonal sera against σ^S^ and CsgD (custom-made by Pineda-Antikörper-Service, Berlin) or a monoclonal antibody against Gfp (Roche), goat anti-rabbit and anti-mouse IgG alkaline phosphatase conjugate (Sigma) and a chromogenic substrate (BCIP/NBT; Boehringer Mannheim) were used.

### Northern blot analysis

For RNA preparation and Northern blot analysis, cells were grown in LB medium and harvested at an OD_578_ as indicated in the figure legends. TRIZOL reagent (Invitrogen) was used to isolate total RNA according to the manufacturer's protocol. Northern blot analysis was performed as described previously (Papenfort *et al*., 2006) with some changes. Five micrograms of total RNA denatured in Ambion loading dye II (Ambion) was separated on 4.5% polyacrylamide gels containing 7 M Urea and transferred to positively charged Nylon Membranes (Roche) by electro-blotting in a tank blotter (Peqlab). Northern probes were random Dig-labelled PCR fragments generated with primer pairs listed in Table S2 and Dig labelling mix (Roche) according to the manufacturers protocol. The *csgD* probe was complementary to the 5′-end of *csgD* mRNA (nucleotides −148 to +90), and the *rprA* probe was complementary to full-size RprA. Prehybridization and hybridization of membranes with DNA probes was carried out in Dig Easy Hyb (Roche) at 47°C overnight. Membranes were washed twice at 42°C for 5 min in 2× SSC/0.1% SDS and twice in 0.1× SSC/0.1% SDS for 30 min at room temperature. Detection of Dig-labelled DNA probes was performed after blocking in blocking solution (Roche) with Dig anti-Fab fragments (Roche) and CDP Star (Roche) as described (Mika and Hengge, 2005). Signals were visualized on Lumi films (Roche) with an Optimax Typ TR developing machine.

### Microarray analysis

For RNA preparation for microarray analysis, cells were grown in LB at 28°C to an OD_578_ of 4.0. Cell lysis, RNA isolation, DNaseI treatment and phenol/chloroform extraction were as previously described (Weber *et al*., 2005). *E. coli* K-12 microarrays containing 4288 gene-specific 50mer oligonucleotide probes representing the whole *E. coli* genome (MWG, Ebersberg, Germany) were used. Hybridization with Cy3/5-dCTP-labelled cDNA, fluorescence detection and image analysis were as described in the Supplement to Pesavento *et al*. (2008). Each microarray experiment was done at least twice (biological replicates; with a dye swap in cDNA labelling). Genes were considered differentially regulated when signal-to-noise ratios exceeded a factor of three, the sum of median intensity counts was above 200, and relative RNA level differences (ratios) were at least twofold in both of the two independent experiments. The original datasets have been deposited in the Array Express database (accession numbers: E-MEXP-2620 for the RprA regulon, E-MEXP-2797 for the CsgD regulon).

Significantly altered gene expression signals (average of the ratios obtained in independent experiments; Table S1) were ordered by their magnitude, and these clustered data were transformed into a ‘heat map’ by the program Mayday 2.9, which provides a platform for visualization, analysis and storage of microarray data (Dietzsch *et al*., 2006). For visualization (Fig. 8), log_2_ values of the signals were used, with a colour range corresponding to values between −4.5 and +4.5.

### Determination of β-galactosidase activity

β-Galactosidase activity was assayed by use of *o*-nitrophenyl-β-d-galactopyranoside (ONPG) as a substrate and is reported as µmol of *o*-nitrophenol per min per mg of cellular protein (Miller, 1972). Experiments showing the expression of *lacZ* fusions along the entire growth cycle were done at least twice, and a representative experiment is shown.
