# Supplementary material for: Targeting of csgD by the small regulatory RNA RprA links stationary phase, biofilm formation and cell envelope stress in Escherichia coli
Source: Mol Microbiol. 2012 Feb 22;84(1):51–65. doi: 10.1111/j.1365-2958.2012.08002.x (PMC3465796; doi:10.1111/j.1365-2958.2012.08002.x)
Supplement: Supplementary file 1 [file mmi0084-0051-SD1.pdf]

Supplementary Material for:

Targeting of *csgD* by the small regulatory RNA RprA links stationary phase, cell envelope stress and biofilm formation in *Escherichia coli*

Franziska Mika, Susan Busse, Alexandra Possling, Janine Berkholz, Natalia Tschowri, Nicole Sommerfeldt, Mihaela Pruteanu and Regine Hengge

Institut für Biologie, Mikrobiologie, Freie Universität Berlin, 14195 Berlin, Germany

Supplementary Materials and Procedures

Generation of strains with chromosomal knockout mutations

All strains used in this study are derivatives of the *E.coli* K-12 strains W3110 (Hayashi *et al.*, 2006) and MC4100 (Casadaban, 1976). Strains used for analysis of *lacZ* fusions carry Δ*lacU169*. Previously described deletion/insertion mutations are *ydaM::cat* (Weber *et al.*, 2006), *hfq::kan* (*hfq1*:: Ω) (Tsui *et al.*, 1994), and *rcsB::kan* (Tschowri *et al.*, 2009). The newly constructed mutant alleles *csgD::kan, rcsC::cat*, *rcsBC::kan*, and *rprA::kan* are deletion/insertion mutations generated by one-step inactivation according to (Datsenko & Wanner, 2000) using the oligonucleotide primers listed in Table S1 (see below). Mutations were transferred by P1 transduction (Miller, 1972).

Construction of plasmids and lacZ and gfp reporter fusions

The primers used for plasmid constructions are listed in Table S1 (see below). RprA is constitutively overexpressed from vector pRprA (pJV100IA-T4) (Urban & Vogel, 2007). pJV100IA-T4 is a derivative of pZE12-*luc* (Expressys, Germany), a high copy number vector with a ColE1 replicon. Expression of the sRNA is driven by the constitutive P_{LlacO} promoter from position +1. Control vector pJV300 is a pZE12-*luc* derivative expressing a small nonsense transcript of the *rrnB*T1,2 region (Urban & Vogel, 2007).

Point mutations in pRprA were introduced using recombinant PCR (2 step 4-Primer method). Plasmid backbone was amplified with Phusion Polymerase, primer pair pZE12_d-*EcoR*I/pZE12_u_(-1) and pZE12-*luc* as template. The insert was amplified using *rprA*_d_(+1)/ *rprA*_u-*EcoR*I as outside primers and the primer pairs *rprA*_d_(35,36 GG->CC)/*rprA*_u_(35,36 GG->CC) and *rprA*_d_(37-39 AAA->TTT)/*rprA*_u_(37-39 AAA->TTT) as inside primers that introduce the (35,36 GG->CC) and (37-39 AAA->TTT) exchanges in RprA. Inserts were cloned blunt/*EcoR*I into pZE12-*luc*, yielding plasmids pAP31 and pAP32, respectively. Plasmids containing point mutations in RprA antisite Ib were introduced by backbone amplification using using Phusion Polymerase, primers *rprA*_d_(63-66 GCTG-> CGAC)/ *rprA*_u_(63-66 GCTG-> CGAC) and plasmids pRprA (pJV100IA-T4) and pAP31 as templates, giving plasmids pFM126 and pFM127, respectively.

Plasmid $pRprA_{60\text{-}105}$ (pFM121) was generated by backbone amplification using Phusion Polymerase, primers *rprA*_d_(+60)/ pZE12_u_(-1) and plasmid pRprA (pJV100IA-T4) as template, followed by religation of blunt ends, carrying a 5´-phosphate.

Previously described translational *lacZ* reporter fusions in *rpoS* (*rpoS742::lacZ*) (Lange & Hengge-Aronis, 1994), *csgB*, *mlrA*, *yaiC*, and *ydaM* (Weber et al., 2006, Sommerfeldt *et al.*, 2009) were used in this study. All *lacZ* reporter fusions are located in single copy at the *att*(λ) location of the chromosome where they had been integrated using phage λRS45 or λRS74 (Simons *et al.*, 1987).

Gfp reporter fusions to *csgD*, *ydaM* and *mlrA* were constructed using pXG10, a low copy vector with a pSC101 origin of replication. Expression of target-gfp fusions is driven by a constitutive P_{LtetO} promoter (Urban & Vogel, 2007). Fusions were constructed such that transcription starts at the same position as from the natural promoters and downstream fusion joints to *gfp* were after nucleotide +90, +60 and +90 of the coding sequences of *csgD*, *ydaM* and *mlrA*, respectively. Primer pairs used were *csgD-Nsi*I/*csgD-Nhe*I, *ydaM-Nsi*I/*ydaM-Nhe*I and *mlrA-Nsi*I/*mlrA-Nhe*I yielding plasmids pSB25, pSB30 and pSB24, respectively. pXG-0 is the corresponding low-copy control vector not expressing any Gfp, which is used for

determination of cellular autofluorescence, and pXG-1 is a similar control vector expressing Gfp only (Urban & Vogel, 2007).

5´-deletions in the *csgD* 5´-UTR/TIR on the plasmid carring the *csgD-148/+90::gfp* fusion (pSB25) were introduced by subcloning fragments of the *csgD* 5´UTR into pXG10 using the primers listed in Table S1 (see below). Primer pairs *csgD*_d_(-36)-*Nsi*I/*csgD-Nhe*I and *csgD*_d_(-120)-*Nsi*I/*csgD-Nhe*I were used to amplify inserts for plasmids pSB29 and pSB27, respectively. Vector pXG10 and inserts were *Nsi*I/*Nhe*I treated and ligated. Primers *csgD*_d_(-83)-*Nsi*I, *csgD*_d_(-103)-*Nsi*I and *csgD*_d_(-111)-*Nsi*I were used each in combination with primer pXG10_u_(-1)-*Nsi*I and pSB25 as template to amplify backbones of plasmids pFM112, pFM117 and pFM118 with Phusion Polymerase, respectively. These plasmid backbones were *Dpn*I and *Nsi*I treated and religated.

Internal deletions in the *csgD* 5´-UTR/TIR on the plasmid carring the *csgD-148/+90::gfp* fusion (pSB25) were introduced using recombinant PCR with 2 overlapping oligonucleotides or blunt end religation of PCR amplified plasmid backbones. pFM113 backbone was amplified using Phusion Polymerase, the overlapping primer pair *csgD*_d_(Δ-87 to -96)/ *csgD*_u_(Δ-87 to -96) and pSB25 as template. pFM116 and pFM120 backbones were amplified using the primer pairs *csgD*_d_(Δ-87 to -119)/ *csgD*_u_(Δ-87 to -119) and *csgD*_d_(Δ-29 to -82)/*csgD*_u_(Δ-29 to -82), respectively, and pSB25 as template. Plasmid backbones were closed by blunt end religation.

Point mutations in the *csgD* 5´-UTR/TIR were introduced by recombinant PCR (2 step 4-Primer method) as described above. For amplification of inserts on the plasmid carring the *csgD-148/+90::gfp* fusion primer pair *csgD-Nsi*I/*csgD-Nhe*I was used as external oligonucleotides. The internal primer pairs *csgD*_d_(–1,–4 CC->GG)/*csgD*_u_(–1,–4 CC->GG) and *csgD*_d _(-78 to -90 CAGC-> GTCG)/*csgD*_u _(-78 to -90 CAGC-> GTCG) were used to generate plasmids pSB33 and pSB41, respectively. Internal primer pairs *csgD*_d_(–1,–4 CC->GG)/*csgD*_u_(–1,–4 CC->GG) and *csgD*_d_(–7 to –5 TTT->AAA)/*csgD*_u_(–7 to –5 TTT->AAA) in combination with external primers *csgD*_d_(-83)-*Nsi*I /*csgD-Nhe*I were used to generate plasmids pAP34 and pAP35, respectively.

In order to generate *csgD-148/+90::gfp* with mutations in site I and II, pSB33 *csgD-148/+90*(–1,–4 CC->GG)*::gfp* was used as a template and again *csgD-Nsi*I/*csgD-Nhe*I as external primers. Internal primers were *csgD*_d_(Δ-87 to -96)/ *csgD*_u_(Δ-87 to -96) and *csgD*_d _(-78 to -90 CAGC-> GTCG)/*csgD*_u _(-78 to -90 CAGC-> GTCG), yielding plasmids pAP36 and pAP37, respectively.

Point mutations in the *ydaM* ORF were introduced by backbone amplification using Phusion Polymerase, primers *ydaM*_d_(12,13CA->GT,18 C->G)/ *ydaM*_u_(12,13CA->GT,18 C->G) and plasmid pSB30 as template, giving plasmid pFM143.

Supplementary Tables

Table S1. The RprA and CsgD regulons determined during entry into stationary phase.[1]

**Name**	**b-number**	**Ratio $rcsC^-$ vs. $rcsC^-$ $rprA^-$**	**Ratio $csgD^+$ vs. $\Delta csgD$**	**Description**
**Negative regulation by RprA / positive regulation by CsgD:**				
argD	b3359	0,321	2.722	subunit of N-succinyldiaminopimelate-aminotransferase/acetylornithine transaminase
artQ	b0862	0,120	5.158	arginine transport system, permease protein
csgA	b1042	0,106	32.530	curli major component
csgB	b1041	0,066	13.502	curli minor component
csgD	b1040	0,068	12.752[2]	regulator for curli and cellulose synthesis
csgE	b1039	0,177	3.008	curli production assembly/transport component
csgF	b1038	0,190	3.308	curli production assembly/transport component
csgG	b1037	0,423	1.922	curli production assembly/transport component
dgoA	b3692	0,280	4.445	2-oxo-3-deoxygalactonate 6-phosphate aldolase and galactonate dehydratase
dnaE	b0184	*1.685*	3.777	DNA polymerase III, alpha subunit
fliI	b1941	0,355	2.798	flagellum-specific ATP synthase
ftsW	b0089	0,129	7.240	membrane protein involved in cell division and shape determination
fxsA	b4140	0,262	3.288	inner membrane protein
gidA	b3741	0,308	*1.798*	protein involved in a tRNA modification pathway
lrp	b0889	0,230	*1.713*	transcriptional regulator and nucleoid component
mdlB	b0449	0,268	*1.833*	predicted multidrug transporter subunit of ABC superfamily: ATP binding component
mnmA	b1133	0,210	4.656	(5-methylaminomethyl-2-thiouridylate)-methyltransferase
nagE	b0679	0,203	3.752	PTS system, N-acetylglucosamine-specific enzyme IIABC
rluC	b1086	0,132	4.141	23S rRNA pseudouridine synthase
rtcB	b3421	0,304	2.375	conserved protein

[1] *E.coli* K-12 strain W3110 as well as its derivatives carrying *rcsC*::*cat* (resulting in hyperactivation of RcsB and increased expression of RprA), *rcsC*::*cat rprA*::*kan* (hyperactivated RcsB, but no RprA) or Δ*csgD* were grown in LB at 28°C to an OD_{578} of 4 and genome-wide transcriptome analysis was performed as detailed in Material and Methods. Ratios of differential gene expression in the strain combinations as indicated are the average of two or more independent experiments and considered significant when > 2.0 or < 0.5. *All* genes for which significant ratios of differential expression were observed for only one or both regulators, are listed (with the ratios considered non-significant shown in grey and italics).

[2] This high ratio for *csgD* itself is due to the Δ*csgD* mutation, ratios observed for *csgE, csgF*, and *csgG* are due to polarity of the Δ*csgD* mutation within the *csgDEFG* operon.

solA	b1059	0,429	*0.725*	N-methyltryptophan oxidase
sthA (udhA)	b3962	0,105	8.708	subunit of pyridine nucleotide transhydrogenase
tolQ	b0737	*0.588*	12.088	Inner membrane protein, part of the Tol-Pal system involved in cell envelope integrity and group A colicin uptake
tolR	b0738	0,068	57.166	Inner membrane protein, part of the Tol-Pal system involved in cell envelope integrity and group A colicin uptake
wcaI	b2050	0,107	6.611	putative colanic biosynthesis glycosyl transferase
yaaX	b0005	*1.101*	3.532	orf, hypothetical protein
ybjK	b0846	0,109	8.830	putative DeoR-like transcriptional regulator
ycfQ	b1111	0,199	7.032	putative DeoR-like transcriptional regulator
ycfT	b1115	0,138	5.857	orf, hypothetical protein
yihV	b3883	0,325	*0.777*	predicted sugar kinase
ymfE	b1138	0,066	60.872	orf, hypothetical protein
yneG	b1523	0.294	4.517	orf, hypothetical protein
yohL	b2105	0,318	2.923	RcnR, transcriptional repressor
yphD	b2546	0,295	*1.819*	subunit of YphD/YphE/YphF ABC transporter
**Positive regulation by RprA / negative regulation by CsgD:**				
dsbC	b2893	2,415	*0.990*	subunit of disulfide isomerase/disulfide oxireductase
gadB	b1493	12,828	*0.620*	glutamate decarboxylase isozyme
gadC	b1492	6,682	0.387	GABA/glutamate transporter
gadE	b3512	7,362	0.492	transcriptional activator
gltF	b3214	3,247	*0.566*	regulator of gltBDF operon, induction of Ntr enzymes
glyS	b3559	3,100	*0.891*	glycine tRNA synthetase, beta subunit
hdeA	b3510	2,582	*0.827*	acid-resistance protein, possible chaperone
hdeB	b3509	2,878	*0.655*	acid stress chaperone
hdeD	b3511	2,122	*0.620*	acid-resistance membrane protein
rpsS	b3316	2,405	*0.912*	30S ribosomal subunit protein S19
tdcC	b3116	2,843	*0.705*	threonine transporter
ygjD	b3064	2,607	*0.841*	predicted peptidase

Table S2. Oligonucleotide primers used in the present study[3].

I. Primers for generating knockout mutations by one-step inactivation[4]:

csgD::kan	5´-AGAGGCAGCTGTCAGGTGTGCGATCAATAAAAAAAGCGGGGTTTC ATC**GTGTAGGCTGGAGCTGCTTC**-3´<br>5´-CCTGCGGCGAACAGAAATTCTGCCGCCACAATCCAGCGTAAATAA CGTTT**CATATGAATATCCTCCTTAG**-3´
rcsC::cat	5´-CATCTGGCATTTGCACTGAATGCCGGATGCGGCGTAAACG **GTGTAGGCTGGAGCTGCTTC**-3´<br>5´-TTTGAAATACCTTGCTTCTTTTCGTACAACCCTGAAAGCC **CATATGAATATCCTCCTTAG**-3´
rcsBC::kan	5´-CAGTTATGTCAAGAGCTTGCTGTAGCAAGGTAGCCTATTAC **GTGTAGGCTGGAGCTGCTTC**-3´<br>5´-TTTGAAATACCTTGCTTCTTTTCGTACAACCCTGAAAGCC **CATATGAATATCCTCCTTAG**-3´
rprA::kan	5´-ATCGACGCAAAAAGTCCGTATGCCTACTATTAGCTCACGG **GTGTAGGCTGGAGCTGCTTC**-3’<br>5´-GGAAAGAGTGAGGGGCGAGGTAGCGAAGCGGAAAAATGTT **CATATGAATATCCTCCTTAG**-3’

II. Primers for cloning $RprA_{60\text{-}105}$ into pFM121 (yielding $pRprA_{60\text{-}105}$):

*rprA*_d_(+60)	5’P-ATTGCTGTGTGTAGTCTTTGC-3’
pZE12_u_(-1)	5’-GTGCTCAGTATCTTGTTATCCG-3’

III. Primers for generating *gfp* gene fusions on pXG-10:

*csgD-Nsi*I	5’-GTTTTT**ATGCAT**CAGATGTAATCCATTAGTTTTATATTTT-3’
*csgD-Nhe*I	5’-GTTTTT**GCTAGC**AAGGTGCTGCAAGAGAGC-3’
*ydaM-Nsi*I	5’-GTTTTT**ATGCAT**GAATTATCTGATCATATGACGTGG-3’
*ydaM-Nhe*I	5’-GTTTTT**GCTAGC**CGAAACGATCCAGACAGGACT-3’
*mlrA-Nsi*I	5’-GTTTTT**ATGCAT**CGGGACCTCGCGAGC-3’
*mlrA-Nhe*I	5’-GTTTTT**GCTAGC**CAGCAATCCGTAACGCCTC-3’

IV. Primers for generating deletion and point mutations in *csgD-148/+90::gfp* (pSB25), in *csgD-83/+90*::*gfp* (pFM112) or in *csgD-148/+90*(−1,−4 CC->GG)::*gfp* (pSB33):

	**5´deletions**	plasmid
*csgD*_d_(-36)-*Nsi*I	5’-GTTTTT**ATGCAT**CAGGTGTGCGATCAATAAAA-3’	pSB29
*csgD*_d_(-83)-*Nsi*I	5’-GTTTTT**ATGCAT**GCAACATCTGTCAGTACTTCTG-3’	pFM112
*csgD*_d_(-103)-	5’-GTTTTT**ATGCAT**TTATTACTACACACAGCAGTGCA-3’	pFM117

---

[3] Unless otherwise indicated, restriction sites are underlined/bold and point mutations are in bold, 5´-phosphate is indicated as 5´P.

[4] Sequences indicated in boldface correspond to sequences present on the resistance cassette plasmids described by Datsenko, K. A. & B. L. Wanner, (2000) One-step inactivation of chromosomal genes in *Escherichia coli* K-12 using PCR products. *Proc. Nat. Acad. Sci. USA* **97**: 6640-6645., the rest of the oligonucleotide primer corresponds to chromosomal DNA sequences around the respective gene.

*Nsi*I		
*csgD*_d_(-111)-*Nsi*I	5'-GTTTTT**<u>ATGCAT</u>**GGGCTGATTTATTACTACACACAG-3'	pFM118
*csgD*_d_(-120)-*Nsi*I	5'-GTTTTT**<u>ATGCAT</u>**ACCCATTTAGGGCTGATTTA-3'	pSB27
pXG10_u_(-1)-*Nsi*I	5'-GTTTTTTT**<u>ATGCAT</u>**GTGCTCAGTATCTCT-3'	pFM112
	**Internal deletions**	
*csgD*_d_(D-87 to -96)	5'-CTGATTTATTACAGTGCAACATCTGTCAGTACTTC-3'	pFM113, pAP36
*csgD*_u_(D-87 to -96)	5'-AGATGTTGCACTGTAATAAATCAGCCCTAAATGG-3'	pFM113, pAP36
*csgD*_d_(D-87 to -119)	5'-TTAGTTTTATATTTTAAGTGCAACATCTGTCAGTAC TTC-3'	pFM116
*csgD*_u_(D-87 to -119)	5'-AGATGTTGCACTTAAAATATAAAACTAATGGATTAC ATCTG-3'	pFM116
*csgD*_d_(D-29 to -82)	5'-CACTGCTGTGTGTAGTAATAAATCAG-3'	pFM120
*csgD*_u_(D-29 to -82)	5´P-CGATCAATAAAAAAAGCGG-3'	pFM120
	**Point mutations**	
*csgD*_d_(-1,-4 CC->GG)	5'-GCGGGGTTT**G**AT**G**ATGTTTAATGAAG-3'	pSB33, pAP34
*csgD*_u_(-1,-4 CC->GG)	5'-CTTCATTAAACAT**C**AT**C**AAACCCCGC-3'	pSB33, pAP34
*csgD*_d_(-7 to -5 TTT->AAA)	5'-GCGGGG**AAA**CATCATGTTTAATGAAG-3'	pAP35
*csgD*_u_(-7 to -5 TTT->AAA)	5'-CTTCATTAAACATGATG**TTT**CCCCGC-3'	pAP35
*csgD*_d _(-78 to -90 CAGC-> GTCG)	5'-ATTTATTACTACACA**GTCG**AGTGCAAC-3'	pSB41, pAP37
*csgD*_u_(-87 to -90 CAGC-> GTCG)	5'-GTTGCACT**CGAC**TGTGTAGTAATAAAT-3'	pSB41, pAP37
*csgD-Nsi*I	5'-GTTTTT**<u>ATGCAT</u>**CAGATGTAATCCATTAGTTTTATATTT T-3'	
*csgD-Nhe*I	5'-GTTTTT**<u>GCTAGC</u>**AAGGTGCTGCAAGAGAGC-3'	

V. Primers for generating point mutations in *ydaM* on pSB30:

*ydaM*_d_(12,13C A->GT,18 C->G)	5'-ATGATTACGCA**<u>GT</u>**ACTT**<u>G</u>**AATACCCTGGACTTACTCACCAGT-3'	pFM143
*ydaM*_u_(12,13C A->GT,18 C->G)	5'-CAGGGTATT**<u>C</u>**AAGT**<u>AC</u>**TGCGTAATCATTGAGATCCC-3'	pFM143

VI. Primers for generating point mutations in *rprA* on pRprA:

*rprA*_d_(35,36	5'-TTTATAAGCAT**CC**AAATCCCCT-3'	pAP31

GG->CC)		
*rprA*_u_(35,36 GG->CC)	5'-AGGGGATTT**GG**ATGCTTATAAA-3'	pAP31
*rprA*_d_(37-39 AAA->TTT)	5'-TTTATAAGCATGG**TTT**TCCCCT-3'	pAP32
*rprA*_u_(37-39 AAA->TTT)	5'-AGGGGA**AAA**CCATGCTTATAAA-3'	pAP32
*rprA*_d_(+1)	5'P-ACGGTTATAAATCAACATATTG-3'	pAP31, pAP32
*rprA*_d_(63-66 GCTG-> CGAC)	5'-CAACGAATT**CGAC**TGTGTAGTCTTTGCCCATCT-3'	pFM126, pFM127
*rprA*_u_(63-66 GCTG-> CGAC)	5'-GACTACACA**GTCG**AATTCGTTGTTTCACTCAGG-3'	pFM126, pFM127
*rprA*_u-*Eco*RI	5'-CG**GAATTC**TAAAAAAAAGCCCATCGT-3'	pAP31, pAP32
pZE12_d-*Eco*RI	5'-CGCACTGACC**GAATTC**ATTAA-3'	pAP31, pAP32
pZE12_u_(-1)	5'-GTGCTCAGTATCTTGTTATCCG-3'	pAP31, pAP32

VII. Primers for generating probes for Northern blot analyses:

*csgD*_d	5'-CAGATGTAATCCATTAGTTTTATATTTT-3'
*csgD*_u	5'-AAGGTGCTGCAAGAGAGC-3'
*rprA*_d	5'-ACGGTTATAAATCAACATATTG-3'
*rprA*_u	5'-TAAAAAAAAGCCCATCGT-3'
5S rRNA_d	5'-TGCCTGGCAGTTCCCTACT-3'
5S rRNA_u	5'-TGCCTGGCGGCAGTAG-3'
*gfp*_d	5'-GAAGGTGATGCAACATACGG-3'
*gfp*_u	5'-AATATAGTTCTTTCCTGTACATAACC-3'

Supplementary Figures

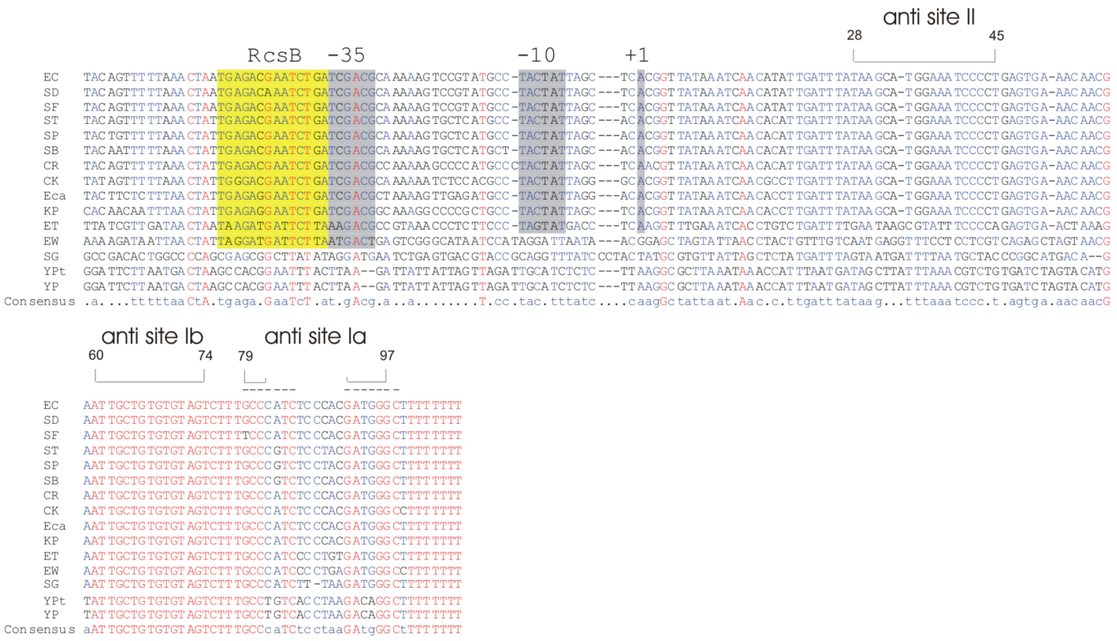

EC: *Escherichia coli*, **SD**: *Shigella dysenteriae*, **SF**: *Shigella flexneri*, **ST**: *Salmonella typhimurium*, **SP**: *Salmonella paratyphi*, **SB**: *Salmonella bongori*, **CR**: *Citrobacter rodentium*, **CK**: *Citrobacter koseri*, **Ecanc**: *Enterobacter cancerogenes*, **KP**: *Klebsiella pneumoniae*, **ET**: Erwinia tasmaniensis, **EW**: *Erwinia carotovora*, **SG**: *Sodalis glossinidius*, **YPt**: *Yersinia pseudotuberculosis*, **YP**: *Yersinia pestis*

Figure S1. **Sequence alignment of *rprA* genes of *Escherichia coli* and other γ-proteobacteria.**
The conserved RcsB binding site, the promoter and the transcriptional start site (where conserved in comparison to *E. coli*) are shown in green, yellow and grey, respectively. Highly conserved nucleotides of *rprA* are shown in red. Above the *E. coli rprA* sequence the anti-Ia, anti-Ib and anti-II regions are indicated that are complementary to regions Ia/Ib and II in *csgD* mRNA. Note that *S. glossinidius*, *Y. pseudotuberculosis* and *Y. pestis* which do not feature a putative RcsB binding site followed by a promoter sequence in their *rprA* regions (last three lines in the alignment), also do not possess a CsgD protein.

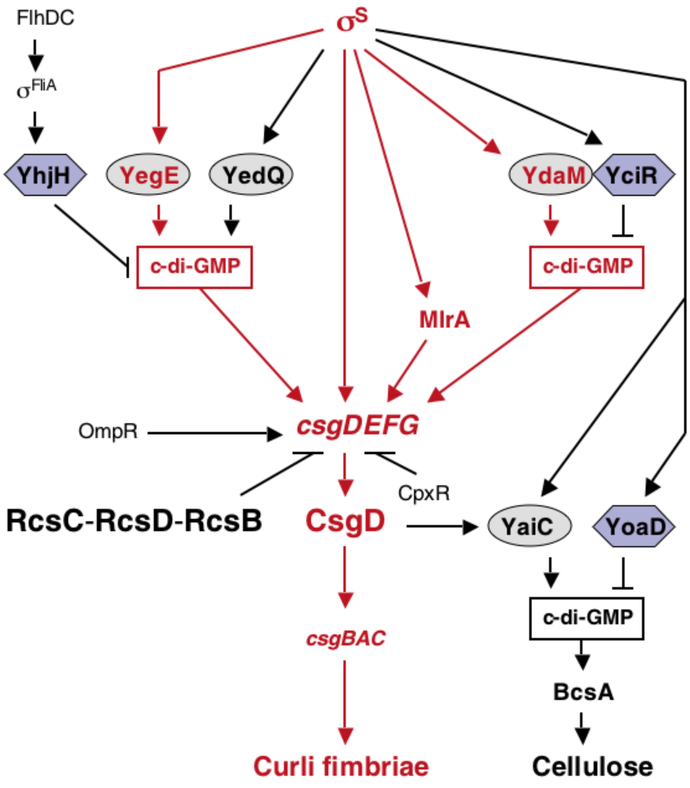

Figure S2. **The 'curli control cascade' in *Escherichia coli*.**
The expression of adhesive curli fimbriae is under the control of a multiple feedforward regulatory cascade depending on the master regulator σ^S, i.e. the general stress response and stationary phase sigma factor (for a recent review, see (Hengge, 2009)). σ^S-controlled components essential for curli expression are (i) the two diguanylate cyclases YdaM and YegE and the second messenger cyclic-di-GMP produced by them (the diguanylate cyclase YedQ has minor modulatory effect only) (Pesavento *et al*., 2008, Weber et al., 2006), and (ii) the MerR-like transcription factor MlrA (Brown *et al*., 2001). These components are involved in transcription from the *csgD* promoter, which is itself a target for σ^S-containing RNA polymerase (Weber et al., 2006). The transcription factor CsgD is required for the activation of the *csgBA* operon which encodes the curli structural proteins. Additional factors required for the assembly of curli fimbriae are encoded by *csgE*, *csgF*, and *csgG* which are located in an operon together with *csgD* (Barnhart & Chapman, 2006, Hammar *et al*., 1995, Römling, 2005). The response regulator OmpR plays a positive modulatory role for transcription from the *csgD* promoter (Gerstel *et al*., 2003, Prigent-Combaret *et al*., 2001). Inhibitory components are the c-di-GMP degrading phosphodiesterases YciR and YhjH, which specifically counteract the activities of YdaM and YegE, respectively (Pesavento et al., 2008, Weber et al., 2006), as well as the two-component response regulators CpxR and RcsB which interfere with CsgD expression (Prigent-Combaret et al., 2001, Tschowri et al., 2009, Vianney *et al*., 2005). Another target for CsgD regulation is *yaiC* (termed *adrA* in *Salmonella*) which encodes yet another diguanylate cyclase that stimulates cellulose biosynthesis (Brombacher *et al*., 2003, Römling *et al*., 2000). Also the *yaiC* gene is under direct (via σ^S-containing RNA polymerase) as well as indirect (via CsgD) positive control by σ^S (Weber et al., 2006). Components and pathways that are essential for the expression of curli fimbriae are shown in red, diguanylate cyclases and phosphodiesterases are indicated with ovoid and hexagonal shapes, respectively.

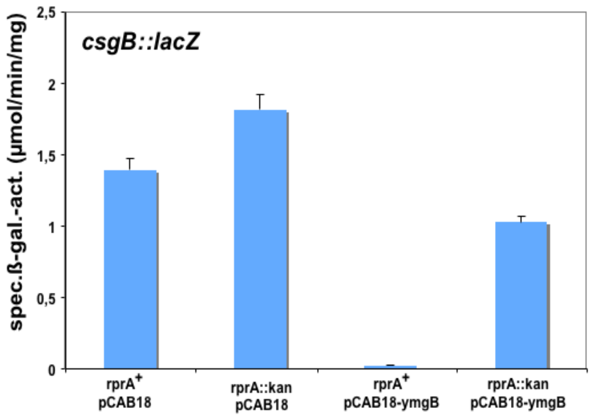

Figure S3. **Mutation of *rprA* suppresses YmgB/RcsB-dependent down-regulation of curli expression.**

E. coli K-12 carrying *csgB::lacZ* (which reflects curli gene expression) and either the low copy number vector pCAB18 or its derivative pYmgB (carrying the *ymgB* gene under *tac* promoter control, described by (Tschowri et al., 2009)) as well as the respective *rprA*::*kan* derivatives were grown in LB/ampicilline at 28ºC. No inducer was added, as the *tac* promoter on pYmgB is sufficiently leaky to allow enough YmgB production to completely repress *csgB::lacZ* expression in an RcsB-dependent manner (Tschowri et al., 2009). Specific β-galactosidase activities were measured in overnight cultures.

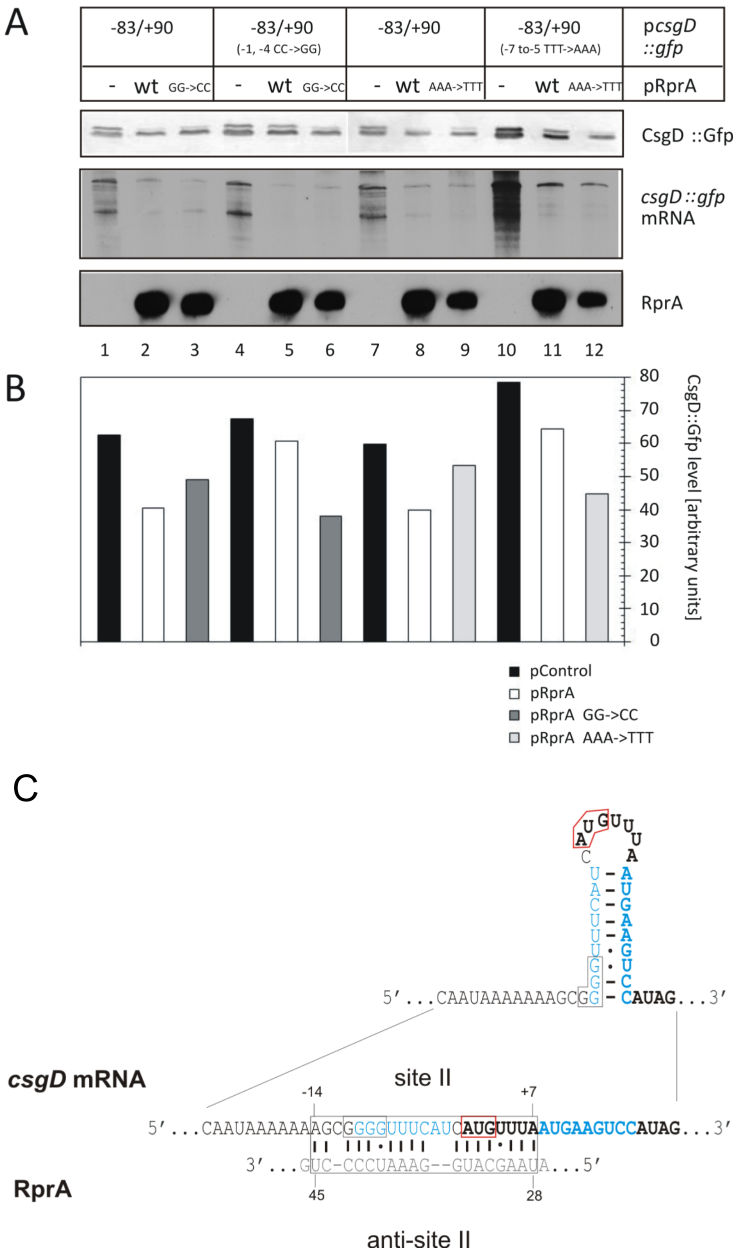

Figure S4. **Effects of point mutations in region II of *csgD* mRNA are reversed by compensatory exchanges in RprA.**

A: An *rprA* mutant derivative of strain MC4100 was transformed with the *csgD-83/+90::gfp* fusion plasmid as well as the RprA expressing plasmids or the corresponding vector (-). Where indicated, these plasmids also carried compensatory point mutations in the *csgD-83/+90::gfp* fusion and/or RprA. Cells were grown in LB/ampicilline/chloramphenicol at 37ºC to an OD_{578} of 4.0. CsgD::Gfp fusion protein was detected by immunoblotting, *csgD::gfp* mRNA, RprA and 5S rRNA were detected by Northern blot analysis.

B: Densitometric quantification of the CsgD::Gfp fusion protein levels shown in A (in the same order of samples).

C: Structure of a small stem-loop that may form in the translational intiation region of *csgD* mRNA. Disruption of this putative stem-loop structure by the TTT(-5to-7)AAA exchange results in increased CsgD::Gfp and *csgD::gfp* mRNA levels (see A).

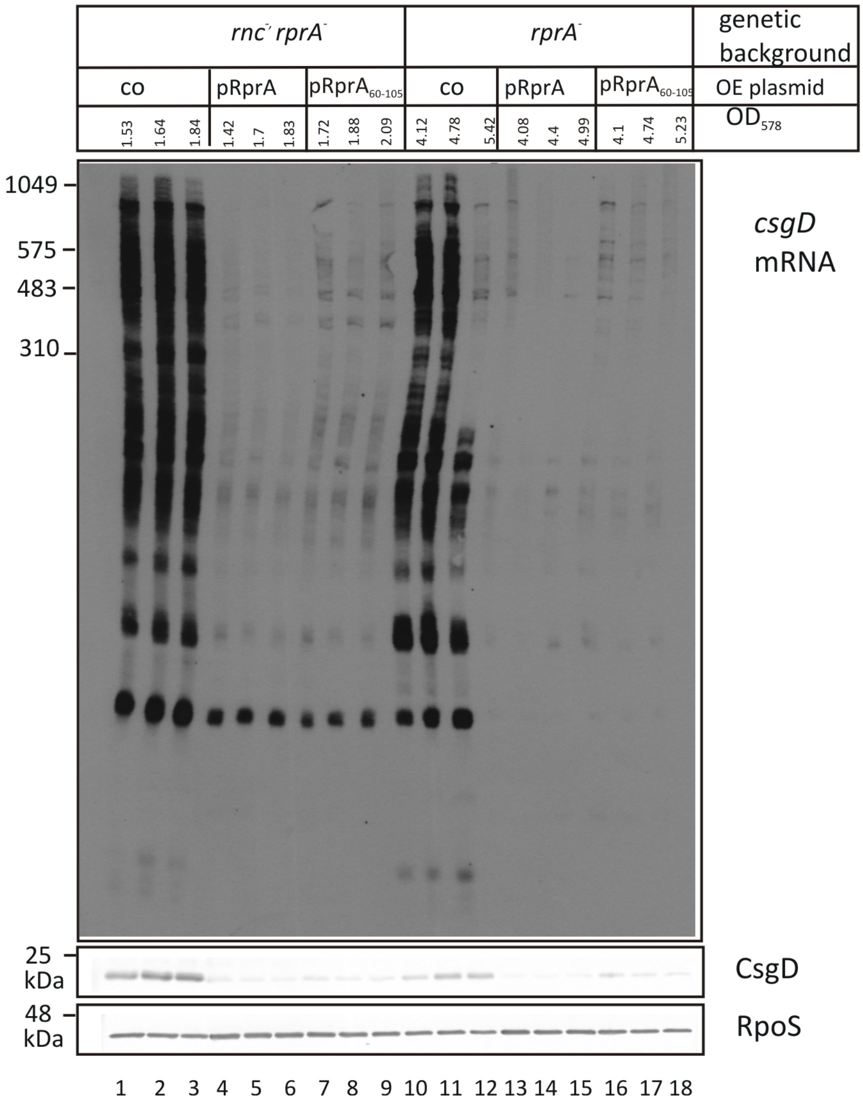

Figure S5. **RprA-mediated down-regulation of *csgD* mRNA does not depend on RNase III.**

The *rnc* mutant BL321 as well as the otherwise isogenic *rnc*$^+$ strain BL322 carrying either the plasmid (pJV300; co), pRprA or pRprA60-105 were grown in LB at 28°C. Since the *rnc* mutant enters into stationary phase and therefore also expresses *csgD* already at an OD_{578} > 1.3, samples were taken during this earlier transition phase. *csgD* mRNA was detected by Northern blot analysis with a probe complementary to the 5´-region of *csgD* mRNA, CsgD and RpoS protein levels were analyzed by immunoblot analysis.

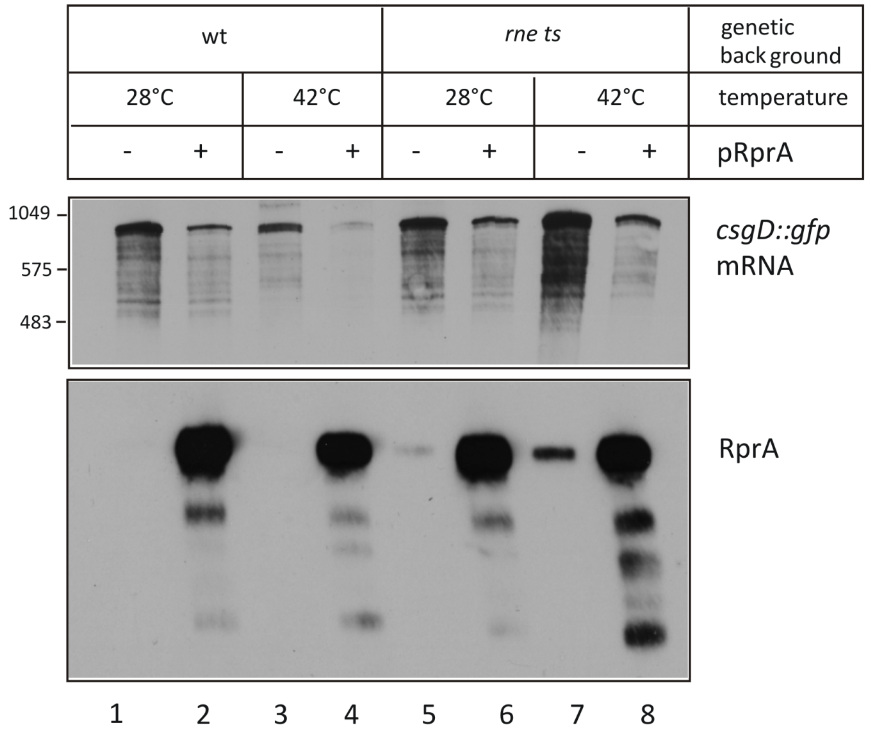

Figure S6. **RNase E is not essential for down-regulation of *csgD* mRNA by RprA.**
The *rne-ts* mutant N3433 expressing a temperature sensitive allele of *rne* as well as the otherwise isogenic rne^{+} strain N3431 carrying the *csgD::gfp* plasmid together with either the control plasmid (pJV300; co) or pRprA were grown in LB at 28°C. Samples were taken 20 min after a temperature upshift to 42 °C at OD_{578} of 2. *csgD::gfp* mRNA and RprA were detected by Northern blot analysis with probes complementary to *gfp* and RprA.

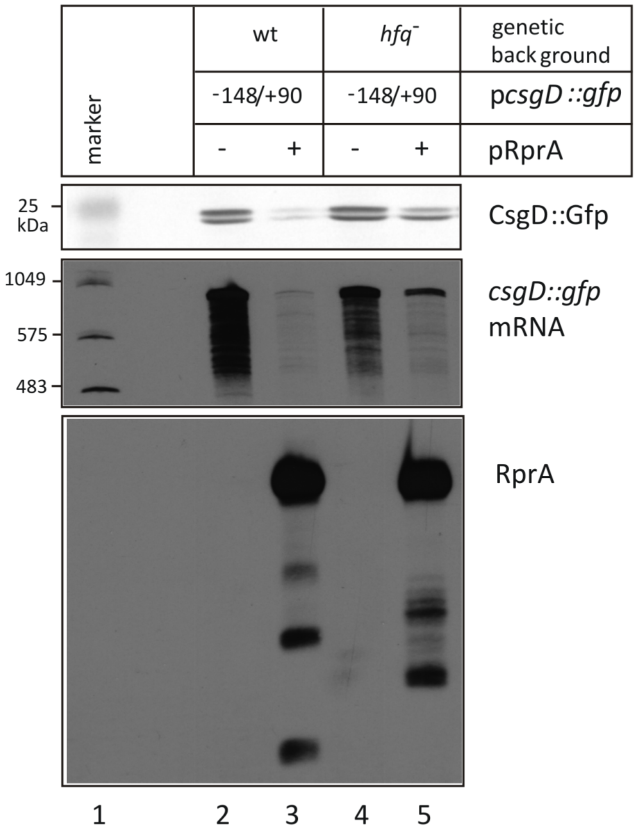

Figure S7. **Down-regulation of *csgD* mRNA by RprA is only slightly affected by the absence of Hfq.**

MC4100 and a *hfq* mutant derivative carrying the *csgD::gfp* plasmid together with pRprA or a control plasmid (pJV300) were grown in LB at 37°C to an OD_{578} of 4.0. RprA, *csgD*::*gfp* mRNA and RprA were determined by Northern with probes complementary to *gfp* and RprA and immunoblot analyses. CsgD::Gfp levels were determined by immunoblot analysis.

Supplementary References

Barnhart, M. M. & M. R. Chapman, (2006) Curli biogenesis and function. *Annu. Rev. Microbiol.* **60**: 131-147.

Brombacher, E., C. Dorel, A. J. B. Zehnder & P. Landini, (2003) The curli biosynthesis regulator CsgD co-ordinates the expression of both positive and negative determinants for biofilm formation in *Escherichia coli*. *Microbiology* **149**: 2847-2857.

Brown, P. K., C. M. Dozois, C. A. Nickerson, A. Zuppardo, J. Terlonge & R. Curtiss III, (2001) MlrA, a novel regulator of curli (Agf) and extracellular matrix synthesis by *Escherichia coli* and *Salmonella enterica* serovar typhimurium. *Mol. Microbiol.* **41**: 349-363.

Casadaban, M. J., (1976) Transposition and fusion of the *lac* genes to selected promoters in *Escherichia coli* using bacteriophage lambda and Mu. *J. Mol. Biol.* **104**: 541-555.

Datsenko, K. A. & B. L. Wanner, (2000) One-step inactivation of chromosomal genes in *Escherichia coli* K-12 using PCR products. *Proc. Nat. Acad. Sci. USA* **97**: 6640-6645.

Gerstel, U., C. Park & U. Römling, (2003) Complex regulation of *csgD* promoter activity by global regulatory proteins. *Mol. Microbiol.* **49**: 639-654.

Hammar, M., A. Arnquist, Z. Bian, A. Olsén & S. Normark, (1995) Expression of two *csg* operons is required for production of fibronectin- and Congo red-binding curli polymers in *Escherichia coli* K-12. *Mol. Microbiol.* **18**: 661-670.

Hayashi, K., N. Morooka, Y. Yamamoto, K. Fujita, K. Isono, S. Choi, E. Ohtsubo, T. Baba, B. L. Wanner, H. Mori & T. Horiuchi, (2006) Highly accurate genome sequences of *Escherichia coli* K-12 strains MG1655 and W3110. *Mol. Syst. Biol.* **2**: 2006.0007.

Hengge, R., (2009) Principles of cyclic-di-GMP signaling. *Nature Rev. Microbiol.* **7**: 263-273.

Lange, R. & R. Hengge-Aronis, (1994) The cellular concentration of the σ^S subunit of RNA-polymerase in *Escherichia coli* is controlled at the levels of transcription, translation and protein stability. *Genes Dev.* **8**: 1600-1612.

Miller, J. H., (1972) *Experiments in molecular genetics*. Cold Spring Harbor Laboratory, Cold Spring Harbor, N. Y.

Pesavento, C., G. Becker, N. Sommerfeldt, A. Possling, N. Tschowri, A. Mehlis & R. Hengge, (2008) Inverse regulatory coordination of motility and curli-mediated adhesion in *Escherichia coli*. *Genes Dev.* **22**: 2434-2446.

Prigent-Combaret, C., E. Brombacher, O. Vidal, A. Ambert, P. Lejeune, P. Landini & C. Dorel, (2001) Complex regulatory network controls initial adhesion and biofilm formation in *Escherichia coli* via regulation of the *csgD* gene. *J. Bacteriol.* **2001**: 7213-7223.

Römling, U., (2005) Characterization of the rdar morphotype, a multicellular behaviour in Enterobacteriaceae. *Cell. Mol. Life Sci.* **62**: 1234-1246.

Römling, U., M. Rohde, A. Olsén, S. Normark & J. Reinköster, (2000) AgfD, the checkpoint of multicellular and aggregative behaviour in *Salmonella typhimurium* regulates at least two independent pathways. *Mol. Microbiol.* **36**: 10-23.

Simons, R. W., F. Houman & N. Kleckner, (1987) Improved single and multicopy *lac*-based cloning vectors for protein and operon fusions. *Gene* **53**: 85-96.

Sommerfeldt, N., A. Possling, G. Becker, C. Pesavento, N. Tschowri & R. Hengge, (2009) Gene expression patterns and differential input into curli fimbriae regulation of all GGDEF/EAL domain proteins in *Escherichia coli*. *Microbiology* **155**: 1318-1331.

Tschowri, N., S. Busse & R. Hengge, (2009) The BLUF-EAL protein YcgF acts as a direct anti-repressor in a blue light response of *E. coli*. *Genes Dev.* **23**: 522-534.

Tsui, H.-C. T., H.-C. L. Leung & M. E. Winkler, (1994) Characterization of broadly pleiotropic phenotypes caused by an *hfq* insertion mutation in *Escherichia coli* K-12. *Mol. Microbiol.* **13**: 35-49.

Urban, J. H. & J. Vogel, (2007) Translational control and target recognition by *Escherichia coli* small RNAs *in vivo*. *Nucl. Acids Res.* **35**: 1018-1037.

Vianney, A., G. Jubelin, S. Renault, C. Dorel, P. Lejeune & J. C. Lazzaroni, (2005) *Escherichia coli tol* and *rcs* genes participate in the complex network affecting curli synthesis. *Microbiology* **151**: 2487-2497.

Weber, H., C. Pesavento, A. Possling, G. Tischendorf & R. Hengge, (2006) Cyclic-di-GMP-mediated signaling within the σ^S network of *Escherichia coli*. *Mol. Microbiol.* **62**: 1014-1034.
